# Impact of nanoparticles on vegetable oil as a cutting fluid with fractional ramped analysis

**DOI:** 10.1038/s41598-023-34344-z

**Published:** 2023-05-02

**Authors:** Faiza Hasin, Zubair Ahmad, Farhad Ali, Naveed Khan, Ilyas Khan, Sayed M. Eldin

**Affiliations:** 1 Department of Mathematics, City University of Science and Information Technology, Peshawar, 25000 Khyber Pakhtunkhwa Pakistan; 2 Department of Mathematics, College of Science Al-Zulfi, Majmaah University, Al-Majmaah, 11952 Saudi Arabia; 3 Center of Research, Faculty of Engineering, Future University in Egypt, New Cairo, 11835 Egypt

**Keywords:** Zoology, Engineering

## Abstract

Better electrical insulation and thermal properties of vegetable oil with nanoparticles are crucial for its uses as a replacement for conventional previous lubricants used in heavy and light industries for cutting and machining. In this study, a magnetohydrodynamic (MHD) flow of a Brinkman-type nanofluid is used to investigate an infinite vertical plate with chemical reaction, heat radiation, and MHD flow. In order to improve the machining and cutting powers of regular vegetable oil, four distinct types of nanoparticles were selected to be the base fluid. The problem is modeled by coupled system partial differential equations (PDEs), and the results are generalized by the Caputo-Fabrizio fractional differential operator for the exponential non-singular kernel. In order to prepare nanofluids, four different types of nanoparticles, namely graphene oxide (GO), molybdenum disulfide (MoS_2_), titanium dioxide (TiO_2_), and aluminum oxide (Al_2_O_3_) are suspended separately in vegetable oil. The results of skin friction, the Nusselt number, and the Sherwood number are computed in various tables. It is found that GO nanoparticles, (followed by MoS_2_, TiO_2_, and Al_2_O_3_) are the materials that can heat transfer at the maximum rate. The heat transfer rate for GO is found to be the greatest with an enhancement up to 19.83% when 4% of nanoparticles are dispersed, followed by molybdenum disulfide at 16.96%, titanium dioxide at 16.25%, and alumina at 15.80%.

## Introduction

Neale et al*.*^1^ investigated the mathematical theory for viscous fluid flow through a porous media. Generally speaking, this law can pass through a porous body with a low permeability. Darcy's law does not apply to some flows that pass through porous surfaces. For fluxes that go across a very permeable medium, the Brinkman model is suitable. The straightforward Navier–Stokes equation cannot be used to analyze the fluid flow behavior in such circumstances. The Brinkman fluid model is one of the models that several academics have presented under certain assumptions. This concept was proposed by Brinkman^2^ for the fluids over a high permeability surface. Consequently, a highly porous material can easily permit the passage of a Brinkman-type fluid. Numerous researchers have used the Brinkman model in their studies after it was created. Convective flows of Brinkman-type fluids are infrequently studied; nonetheless, these studies have a wide range of applications in industry^3,4^. Mass and heat transfer is the main focus of these studies.

Choi was the first to discover the idea of nanofluids by distributing the nanoparticles in common fluids^5^. According to the published work, nanofluids have better thermal diffusivity, viscosity, and thermal conductivity when compared to pure base fluids like water and vegetable oil. These are examples of thermophysical qualities. Numerous studies examined the uses of various nanofluids for heat transfer using theoretical and experimental methods. Two methods can be used to create nanofluids through experimentation. One- or two-step procedures are frequently used to prepare nanofluids. Compared to the one-step method, the two-step strategy is more commonly utilized since it is simpler, however this method makes it more difficult to obtain hybrid nanofluid stability^6–8^. The one-step approach, on the other hand, can maintain relatively high production costs while providing high levels of consistency in nanofluids. Surfactant addition, surface treatment, and ph alteration were some of the techniques explored by several researchers to create a lasting nanofluid. These methods do, however, have significant drawbacks, such as the deterioration of the hybrid nanofluids' thermal properties^9^. Saidur et al*.*^10^ touched on certain difficulties and uses in contemporary industries. Numerous researchers have investigated, produced, and tested various nanofluids for use in heat transfer applications^11–13^.

Nanoparticles' sizes and shapes also affect their thermophysical characteristics. Timofeeva experimentally researched numerous kinds of alumina using ethylene glycol and water as a base fluid^14^. He found that the shape of the nanoparticle affects how much heat can be transferred, and that aluminum oxide, which has a platelet-like shape, had the maximum thermal conductivity and heat transfer rate. In addition to experimental methods, various theoretical strategies have also been used in recent years. The size-dependent characteristics of nanoparticles have been investigated using a number of models^15–17^. Meiorin et al*.*^18^ investigation of magnetic nanoparticle-based super magnetic nanocomposites used vegetable oil as a basis fluid. Recently, research on polyhedral, disk-shaped nanoparticles^19,20^ has also taken place. These published models, meanwhile, fail to effectively capture the impact of particle shape on the properties of non-spherical nanoparticles. Hamilton and Crosser^21^ looked into how different particle morphologies affected thermal conductivities. Khan et al.^22^ explored the numerical study of nanofluid flow on a rotating disc using Buongiorno's model. Li et al.^23^ investigation into the effects of thermal conductivity in fluids containing oxide nanoparticles.

In industrial applications, nanofluids are used in lubricants, heat sinks with microchannels, and heat exchangers. The operation of the magnetic cell, drug administration, hyperthermia, and continuous development of magnetic resonance imaging are some examples of biomedical nanofluid uses. (MRI). Compared to water-based nanofluids, oil-based nanofluids have garnered very little attention in the literature. There is a critical need for oil-based heat transfer fluids with considerably improved thermal conductivity in many industrial fields of science and technology. The type of cutting fluid used can have a major impact on machining performance if it is applied, chosen, and disposed of properly. It is common practice to use traditional lubrication systems during machining to reduce heat and increase cutting power. Groundnut oil, a type of vegetable oil, has been used as a metal-cutting agent in machining operations recently. Fairuz et al*.*^24^ used a variety of lubricants based on vegetable oil to analyze chip generating and tool wear throughout the drilling process. Kuram et al*.*^25^ looked into how drilling affected cutting fluids made of vegetable oil. The passage of thermal energy from one concentration to another is referred to as heat transfer. On the other hand, a mass transfer is the whole transfer of mass from one location to another. Convection, conduction, and radiation are the methods for transmitting heat. It serves a multitude of functions in modern science and technology, including refrigerating, fractional distilling, processing petrochemicals, and purifying crude oil. For the vaporization of water, Khan et al*.*^26^ examined a Maxwell nanofluid flows over an infinite vertical plate with ramping and isothermal wall temperature and concentration. They found that increasing the quantity of nanoparticles for the ramping wall condition increased heat transfer to 12.895 percent, while improving the isothermal wall temperature of regular engine oil to 12.899 percent. They also found that the rate of mass transfer for the ramped wall condition decreased to 3.030 percent, while improving the isothermal wall boundary condition to 3.104 percent.

Chandran et al*.*^27^ used a ramping wall temperature close to the plate to study convection flow. Khalid et al*.*^28^ looked at the free convection flow of nanofluids as the wall temperature increased. Ramped wall temperature settings were utilized by Ghara et al*.*^29^ to investigate the effects of MHD-free convection flow across an oscillating plate. The ramping and isothermal wall temperature with concentration was studied by Hasin et al*.*^30^. When escalating the wall temperature, Haq et al*.*^31^ explored slip conditions in unstable viscous fluid flow. Other ramping and isothermal wall concentration and temperature applications include generator systems, biofluids, and nanofluids; see^32,33^.

Reddy et al*.*^34^ studied the behavior of hybrid nanofluid with the combined effect of heat and mass transfer, thermal radiation and chemical reaction on entropy generation analysis characteristics over a stretching sheet with slip effects. Reddy and Sreedevi^35^ studied the numerical investigation of entropy generation, flow and heat transport characteristics within a closed chamber utilizing a hybrid nanofluid containing silver particles, carbon nanotubes and water as a common fluid is performed in this study using captivating radiation and magnetic fields. A vertical cone filled with nanofluid saturated porous medium is investigated for boundary layer flow characteristics, heat transfer characteristics, and first order chemical reaction during convection was studied by Reddy et al*.*^36^. Numerical simulations are performed to investigate the interaction between MWCNTs, water-based nanofluid flow, isentropic lines and isotherms inside a square enclosure with radiation and magnetic fields has been investigated by Reddy et al*.*^37^. Using a stretching sheet embedded in porous media filled with viscous micropolar fluid, the influence of Soret and Dufour effects is numerically investigated over the unsteady magnetohydrodynamic boundary layer flow was studied by Reddy et al*.*^38^. Heat transfer and flow analysis of water-CNTs type nanofluid between two stretchable revolving disks are numerically investigated in the presence of thermal radiation and magnetic field investigated by Reddy et al*.*^39^ .

Since the advent of integer derivatives, research on fractional-order derivatives has been conducted. This idea has gained a lot of acceptance over the past three decades^40–44^, and it is no longer just applicable to mathematics. Fractional derivatives are the standardization of classical derivatives. As a result of Leibniz's conception of the nth-order derivative, fractional calculus was created. Del Hospital questioned Leibniz over fractional order^45–48^. What would happen? A variety of science, engineering, and technology areas have used the potent mathematical tool of fractional calculus to examine real-world applications. Its uses in fluid mechanics, bioengineering, applied mathematics, signal processing, electrochemistry, physics, finance, and viscoelasticity have lately come to light^48^. Numerous scholars also thought that fractional derivatives may be used to evaluate the Brinkman-type fluid flow. To determine precise solutions for incompressible viscous fluid flows across a porous medium at MHD, Haq et al*.*^49^ looked at the Caputo-Fabrizio fractional operator. Saqib et al*.*^50^ investigated the precise solutions of spontaneous convective flow using the Caputo-Fabrizio operator. To get the precise answer for velocity and temperature, they used the Laplace transform method. Interesting studies on Brinkman-type fluid while dealing with fractional differential operators have also been published by^51,52^.

Based on the literature justification stated above, no one has thought of using fractional derivatives to model the transmission of mass and heat in the MHD flow of a Brinkman-type fluid. This work uses a Brinkman-type nanofluid flow on an infinite vertical plate along with a chemical reaction, thermal radiation, and an MHD flow. Vegetable oil is used as the basis fluid, and four different types of nanoparticles are chosen to enhance the cutting and machining characteristics. Coupled system PDEs are used to describe the issue, and they are then generalized using the Caputo-Fabrizio fractional differential operator for the exponential non-singular kernel. The effective results, which are also represented through various figures and tables and thoroughly discussed, are calculated using the Laplace transform.

## Mathematical formulation

**Figure 1:**
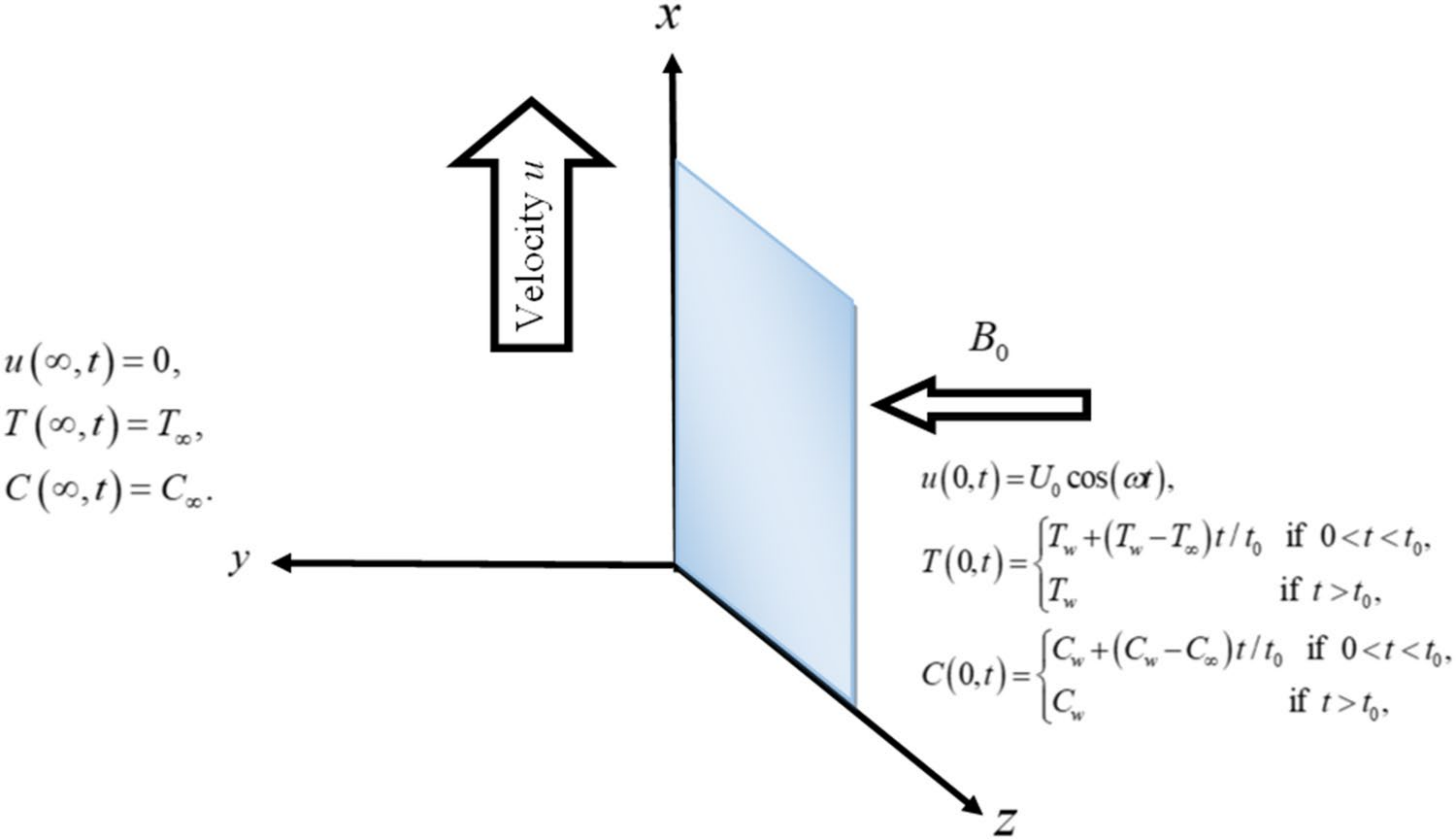
The problem geometry.

In this study, we explore the unstable, laminar, unidirectional, and unidimensional incompressible MHD flow of a Brinkman-type fluid through an infinite plate. The direction of the applied magnetic field is taken to be perpendicular, While the low magnetic Reynold number ignores the generated magnetic field. The fully developed flow is taken along the x-axis. The fluid occupies the space for $y \ge 0$. Initially $t_{1} \le 0$, both the fluid and the plate are at rest with constant concentration $C_{\infty }$ and ambient temperature $T_{\infty }$. For $t_{1} = 0^{ + }$, the plate begins to oscillate in its own plane with frequency $\omega$ and velocity $U_{0}$. Concentration and temperature of the plate is increased to $C_{\infty } + \left( {C_{w} - C_{\infty } } \right)t_{1} /t_{0}$ and $T_{\infty } + \left( {T_{w} - T_{\infty } } \right)t_{1} /t_{0}$ respectively as shown in Fig. 1, which is given below:

The fields for velocity, temperature, and concentration are given below:

1 $$ \left\{ \begin{gathered} \vec{V} = \left( {u_{1} \left( {y_{1} ,t_{1} } \right),0,0} \right), \hfill \\ T = T_{1} \left( {y_{1} ,t_{1} } \right), \hfill \\ C = C\left( {y_{1} ,t_{1} } \right). \hfill \\ \end{gathered} \right. $$

By using Eq. (1), the Brinkman-type nanofluid model becomes^53^:

2 $$ \begin{gathered} \rho_{nf} \left( {\frac{{\partial u_{1} (y_{1} ,t_{1} )}}{{\partial t_{1} }} + \beta *u_{1} (y,t)} \right) = \mu_{nf} \frac{{\partial^{2} u_{1} (y_{1} ,t_{1} )}}{{\partial y_{1}^{2} }} - \sigma_{nf} B_{0}^{2} u_{1} (y_{1} ,t_{1} ) \hfill \\ + g(\rho \beta_{T} ){}_{nf}\left( {T_{1} (y_{1} ,t_{1} ) - T_{\infty } } \right) + g\left( {\rho \beta_{c} } \right)_{nf} \left( {C_{1} \left( {y_{1} ,t_{1} } \right) - C_{\infty } } \right), \hfill \\ \end{gathered} $$

3 $$ (\rho c_{p} )_{nf} \frac{{\partial T_{1} (y_{1} ,t_{1} )}}{{\partial t_{1} }} = k_{nf} \frac{{\partial^{2} T_{1} (y_{1} ,t_{1} )}}{{\partial y_{1}^{2} }} - \frac{{\partial q_{r} }}{{\partial y_{1} }}, $$

4 $$ \frac{{\partial C_{1} \left( {y_{1} ,t_{1} } \right)}}{{\partial t_{1} }} = D_{nf} \frac{{\partial^{2} C_{1} \left( {y_{1} ,t_{1} } \right)}}{{\partial y_{1}^{2} }} - k(C_{w} - C_{\infty } ), $$

where $\rho_{nf}$ is the density, $u_{1}$ Dimensional velocity of the fluid in $x -$ direction, $\mu_{nf}$ is the dynamic viscosity, $\sigma_{nf}$ is the electrical conductivity, $(\beta_{T} )_{nf}$ is the thermal expansion coefficient of nanofluid, $\left( {c_{p} } \right)_{nf}$ is the heat capacitance, $k_{nf}$ is the thermal conductivity, $D_{nf}$ is the thermal diffusivity, $(\beta_{c} )_{nf}$ is the concentration expansion of nanofluid respectively. The Brinkman type fluid parameter is $\beta^{*}$ and $B_{0}$ is the uniform magnetic field, g is the gravitational acceleration, *T* is the fluid temperature, $T_{\infty }$ Ambient temperature, $qr$ is the thermal radiation, $C_{\infty }$ Constant concentration, $C_{1}$ concentration of the fluid, $g$ Acceleration due to gravity $k\left( {C_{w} - C_{\infty } } \right)$ is the chemical reaction, where C stands for concentration. Following are the suitable physical initial and boundary conditions^53^:

5 $$ \left. \begin{gathered} u_{1} \left( {y_{1} ,0} \right) = 0,\,\,\,T_{1} \left( {y_{1} ,0} \right) = T_{\infty } ,\,\,\,C_{1} \left( {y_{1} ,0} \right) = C_{\infty } ,\, \hfill \\ u_{1} \left( {0,t_{1} } \right) = U_{0} \cos \left( {\omega t_{1} } \right), \hfill \\ T_{1} \left( {0,t_{1} } \right) = \left\{ {\begin{array}{*{20}l} {T_{w} + \left( {T_{w} - T_{\infty } } \right)t_{1} /t_{0} } \hfill & {{\text{if }}0 < t_{1} < t_{0} ,} \hfill \\ {T_{w} } \hfill & {{\text{if }}t_{1} > t_{0} ,} \hfill \\ \end{array} } \right. \hfill \\ C_{1} \left( {0,t_{1} } \right) = \left\{ {\begin{array}{*{20}l} {C_{w} + \left( {C_{w} - C_{\infty } } \right)t_{1} /t_{0} } \hfill & {{\text{if }}0 < t_{1} < t_{0} ,} \hfill \\ {C_{w} } \hfill & {{\text{if }}t_{1} > t_{0} ,} \hfill \\ \end{array} } \right. \hfill \\ u_{1} \left( {y_{1} ,t_{1} } \right) = 0,\,\,\,T_{1} \left( {y_{1} ,t_{1} } \right) = T_{\infty } ,\,\,\,C_{1} \left( {y_{1} ,t_{1} } \right) = C_{\infty } ;\,\,y \to \infty \hfill \\ \end{gathered} \right\} $$

The nanofluid expressions for spherical-shaped nanoparticles were given by Oztop and Abu-Nada^54^ and employing different types of nanofluid and topology structures are given in^6,8,55^. The mathematical expressions for $\rho_{nf,}$ $\mu_{nf,}$ $\sigma_{nf}$ $(\beta_{T} )_{nf}$ , $(\beta_{c} )_{nf}$ , $(c_{p} )_{nf,}$ and $k_{nf,}$ are given below.

6 $$ \left. \begin{gathered} D_{nf} = (1 - \phi )D_{f} ,\,(\rho c_{p} )_{nf} = (1 - \phi )(\rho c_{p} )_{f} + \phi (\rho c_{p} )_{s} ,\rho_{nf} = (1 - \phi )\rho_{f} + \phi \rho_{s} ,\, \hfill \\ (\rho \beta_{T} )_{nf} = (1 - \phi )(\rho \beta_{T} )_{f} + \phi (\rho \beta_{T} )_{s} ,\,\,\,(\rho \beta_{C} )_{nf} = (1 - \phi )(\rho \beta_{C} )_{f} + (\rho \beta_{C} )_{S} , \hfill \\ \, \hfill \\ \frac{{k_{nf} }}{{k_{f} }} = \frac{{2k_{f} + k_{s} - 2\phi \left( {k_{f} - k_{s} } \right)}}{{2k_{f} + k_{s} + 2\phi \left( {k_{f} - k_{s} } \right)}},\,\,\,\,\frac{{\sigma_{nf} }}{{\sigma_{f} }} = 1 + \frac{{3\left( {\left. {\frac{{\sigma_{s} }}{{\sigma_{f} }} - 1} \right)} \right.\phi }}{{\left. {\left( {\frac{{\sigma_{s} }}{{\sigma_{f} }} + 2} \right.} \right) - \left( {\left. {\frac{{\sigma_{s} }}{{\sigma_{f} }} - 1} \right)\phi } \right.}},\, \hfill \\ \mu_{nf} = \frac{{\mu_{f} }}{{(1 - \phi )^{2.5} }},\, \hfill \\ \, \hfill \\ \end{gathered} \right\} $$

The notations *nf, f,* and *s* in Eq. (6) are called nanofluid, base fluid, and solid nanoparticles, respectively.

Here are the following variables without dimensions for non-dimensonalization:

7 $$ \left. {u = \frac{{u_{1} }}{{U_{0} }},\,\,\zeta = \frac{{U_{0} }}{{\upsilon_{f} }}y_{1} ,\,\,\tau = \frac{{t_{1} U_{0}^{2} }}{{\upsilon_{f} }},\,\,\theta = \frac{{T_{1} - T_{\infty } }}{{T_{w} - T_{\infty } }},\,\,\psi = \frac{{C_{1} - C_{\infty } }}{{C_{w} - C_{\infty } }}} \right\}. $$

By using Eq. (7) and removing the "*" from the initial and boundary conditions, the governing equations' dimensionless form is as follows:

8 $$ \chi_{0} \left( {\frac{\partial u}{{\partial \tau }} + \beta } \right) = \chi_{1} \frac{{\partial^{2} u}}{{\partial \zeta^{2} }} - \chi_{2} Mu + \chi_{3} Gr\theta + \chi_{4} Gm\psi $$

9 $$ \frac{\partial \theta }{{\partial \tau }} = \frac{1}{{p_{0} }}\frac{{\partial^{2} \theta }}{{\partial \zeta^{2} }} $$

10 $$ \frac{\partial \Phi }{{\partial \tau }} = \frac{{b_{0} }}{sc}\frac{{\partial^{2} \Phi }}{{\partial \zeta^{2} }} - \gamma \Phi $$

with

11 $$ \left. \begin{gathered} u\left( {\zeta ,0} \right) = 0,\,\,\,\theta \left( {\zeta ,0} \right) = 0,\,\,\,\psi \left( {\zeta ,0} \right) = 0,\, \hfill \\ u\left( {0,\tau } \right) = H(\tau )\cos \left( {\omega \tau } \right), \hfill \\ \theta \left( {0,\tau } \right) = \left\{ \begin{gathered} \tau \,\,\,\,{\text{if }}0 < \tau < 1, \hfill \\ 1\,\,\,\,\,\,{\text{if }}\tau > 1,\,\,\,\tau H\left( \tau \right) - \left( {\tau - 1} \right)H\left( {\tau - 1} \right), \hfill \\ \end{gathered} \right. \hfill \\ \psi \left( {0,\tau } \right) = \left\{ \begin{gathered} \tau \,\,\,\,{\text{if }}0 < \tau < 1, \hfill \\ 1\,\,\,\,\,\,{\text{if}}\,\,\tau > 1,\,\,\tau H\left( \tau \right) - \left( {\tau - 1} \right)H\left( {\tau - 1} \right) \hfill \\ \end{gathered} \right. \hfill \\ u\left( {\zeta ,\tau } \right) = 0,\,\,\,\theta \left( {\zeta ,\tau } \right) = 0,\,\,\,\psi \left( {\zeta ,\tau } \right) = 0;\,\,\,\zeta \to \infty . \hfill \\ \end{gathered} \right\} $$

where

$$ \begin{gathered}   \beta  = \frac{{\upsilon _{f} \beta ^{*} }}{{U_{0} ^{2} }},\;M = \frac{{\upsilon _{f} \sigma _{f} B_{0} ^{2} }}{{U_{0} ^{2} \rho _{f} }},\;Gr = \frac{{g(\beta _{T} \upsilon )_{f} (T_{w}  - T_{\infty } )}}{{U_{0} ^{3} }},\;Gm = \frac{{g\beta _{c} (c_{w}  - c_{\infty } )}}{{U_{0} }},\;\Pr  = \left( {\frac{{\mu c_{p} }}{k}} \right)_{f} , \hfill \\   Sc = \left( {\frac{D}{\nu }} \right)_{f} \chi _{0}  = (1 - \phi ) + \phi \frac{{\rho _{s} }}{{\rho _{f} }},\;\chi _{1}  = \frac{1}{{(1 - \phi )^{{2.5}} }},\;\chi _{2}  = 1 + \frac{{3\left( {\left. {\frac{{\sigma _{s} }}{{\sigma _{f} }} - 1} \right)} \right.\phi }}{{\left. {\left( {\frac{{\sigma _{s} }}{{\sigma _{f} }} + 2} \right.} \right) - \left( {\left. {\frac{{\sigma _{s} }}{{\sigma _{f} }} - 1} \right)\phi } \right.}}, \hfill \\   \chi _{3}  = (1 - \phi ) + \phi \frac{{(\rho \beta _{T} )_{s} }}{{(\rho \beta _{T} )_{f} }},\;\chi _{4}  = (1 - \phi ) + \phi \frac{{(\rho C_{p} )_{s} }}{{(\rho C_{P} )_{f} }},\;b_{0}  = \frac{{\left( {1 - \phi } \right)}}{{Sc}},\;\frac{{\partial q_{r} }}{{\partial y_{1} }} =  - \frac{{16\sigma _{1} }}{{3k_{3} }}T_{\infty } ^{3} \frac{{\partial {}^{2}T}}{{\partial y{}^{2}}} \hfill \\   \lambda _{{nf}}  = \frac{{k_{{nf}} }}{{k_{f} }},\;p_{0}  = \frac{{\Pr \chi _{4} }}{{(\lambda _{{nf}}  + Nr)}}\gamma  = kt_{0}  \hfill \\  \end{gathered}  $$

Here, $$\beta ,M,Gr,Gm,\Pr$$ and $$Sc$$ are the parameter of Brinkman-type fluid, magnetic number, thermal and mass Grashof numbers, Prandtl number and Schmidt number respectively. In addition, $$\chi_{0} ,\chi_{1} ,\chi_{2} ,\chi_{3}$$$$\chi_{4}$$,$$b_{0}$$ and p_0_ are constant terms that arise as a result of the calculations. The dimensionless governing Eqs. (8) to (10) are expressed in Caputo–Fabrizio time fractional form as:

12 $$ \begin{gathered} \chi_{0} \left( {{}^{CF}D_{\tau }^{\alpha } u\left( {\zeta ,\tau } \right) + \beta u\left( {\zeta ,\tau } \right)} \right) = \chi_{1} \frac{{\partial^{2} u\left( {\zeta ,\tau } \right)}}{{\partial \zeta^{2} }} - \chi_{2} Mu\left( {\zeta ,\tau } \right) + \hfill \\ \chi_{3} Gr\theta \left( {\zeta ,\tau } \right) + \chi_{4} Gm\psi \left( {\zeta ,\tau } \right), \hfill \\ \end{gathered} $$

13 $$ {}^{CF}D_{\tau }^{\alpha } \theta \left( {\zeta ,\tau } \right) = \frac{1}{{p_{0} }}\frac{{\partial^{2} \theta \left( {\zeta ,\tau } \right)}}{{\partial \zeta^{2} }}, $$

14 $$ {}^{CF}D_{\tau }^{\alpha } \psi \left( {\zeta ,\tau } \right) = b_{0} \frac{{\partial^{2} \psi \left( {\zeta ,\tau } \right)}}{{\partial \zeta^{2} }} - \gamma \psi \left( {\zeta ,\tau } \right) $$

where $^{CF} D_{t}^{\alpha }$ (.) is defined by^56^ as the Caputo-Fabrizio fractional-time operator (CFTO).

15 $$ {}^{CF}D_{\tau }^{\alpha } f\left( {\zeta ,\tau } \right) = \frac{N\left( \alpha \right)}{{\left( {1 - \alpha } \right)}}\int\limits_{0}^{\tau } {f^{\prime}\left( {\zeta ,\tau } \right)\exp \left( { - \frac{\alpha (\tau - t)}{{1 - \alpha }}} \right)} dt;\,\,\,\,0 < \alpha < 1, $$

With N(1) = N(0) = 1, $N(\alpha )$ is the normalization function.

## Solution of the problem

Using the Laplace transform approach, this section offers the exact solutions to the fractional model under consideration.

### Solutions of temperature field

Using the initial and boundary conditions and applying the Laplace transform to Eq. (13)

16 $$ \frac{{d\overline{\theta }\left( {\zeta ,s} \right)}}{{d\zeta^{2} }} - \frac{{sa_{0} p_{0} }}{{s + a_{1} }}\overline{\theta }\left( {\zeta ,s} \right) = 0, $$

with the transform boundary conditions.

17 $$ \overline{\theta }\left( {\zeta ,s} \right) = \int\limits_{0}^{1} {\tau .e^{ - s\tau } } + \int\limits_{1}^{\infty } {1.e^{ - s\tau } } = \frac{{1 - e^{ - s} }}{{s^{2} }},\;{\text{and}}\;\overline{\theta }\left( {\infty ,s} \right) = 0. $$

In the Laplace transform domain, the boundary conditions mentioned in Eq. (17) are applied to get the solutions of Eq. (16):

18 $$ \overline{\theta }\left( {\zeta ,s} \right) = \frac{1}{{s^{2} }}\exp \left( { - \zeta \sqrt {\frac{{sa_{0} p_{0} }}{{s + a_{1} }}} } \right) - \exp \left( { - \zeta \sqrt {\frac{{sa_{0} p_{0} }}{{s + a_{1} }}} } \right), $$

where $$a_{0} = \frac{1}{1 - \alpha }$$ and $$a_{1} = \frac{\alpha }{1 - \alpha }$$.

Equation (18) is it is possible to further simplify the energy equation with ramping wall temperature as follows:

19 $$ \overline{\theta }\left( {\zeta ,s} \right) = \overline{\theta }_{Ramp} \left( {\zeta ,s} \right) - e^{ - s} \overline{\theta }_{Ramp} \left( {\zeta ,s} \right), $$

where

20 $$ \overline{\theta }_{Ramp} \left( {\zeta ,s} \right) = \overline{f}_{Ramp} (\zeta ,s,a_{0} p_{0} ,a_{1} ) = \frac{1}{{s^{2} }}\exp \left( { - \zeta \sqrt {\frac{{sa_{0} p_{0} }}{{s + a_{1} }}} } \right). $$

Equation (19) is transformed back using the inverse Laplace transform, which yield:

21 $$ \theta \left( {\zeta ,\tau } \right) = \theta_{Ramp} \left( {\zeta ,\tau } \right) - \theta_{Ramp} \left( {\zeta ,\tau - 1} \right)H(\tau - 1), $$

where $H(\tau - 1)$ is the Heaviside step function. The term $\theta_{Ramp} \left( {\zeta ,\tau } \right)$ is obtained as:

22 $$ \theta_{Ramp} \left( {\zeta ,\tau } \right) = \int\limits_{0}^{\tau } {f_{Ramp} (\zeta ,t,a_{0} } ,p_{0} ,a_{1} )dt, $$

where

23 $$ f_{Ramp} \left( {\zeta ,\tau ,a_{0} ,p_{0} ,a_{1} } \right) = 1 + \frac{{2a_{0} p_{0} }}{\pi }\int\limits_{0}^{\infty } {\frac{{\sin \left( {\zeta x} \right)}}{{x\left( {a_{0} p_{0} + x^{2} } \right)}}} \exp \left( { - \frac{{a_{1} x^{2} \zeta \tau }}{{a_{0} p_{0} + x^{2} }}} \right)dx. $$

Equation (21) shows the exact energy solutions for a ramping wall temperature, when $0 < t < 1.$ To find the isothermal temperature, solve Eq. (13) for the isothermal temperature condition, which gives:

24 $$ \overline{\theta }_{iso} \left( {\zeta ,s} \right) = \frac{1}{s}\exp \left( { - \zeta \sqrt {\frac{{a_{0} p_{0} s}}{{s + a_{1} }}} } \right), $$

such that

25 $$ \overline{\theta }_{iso} \left( {\zeta ,s} \right) = \overline{g}_{iso} (\zeta ,s,a_{0} p_{0} ,a_{1} ) = \frac{1}{s}\exp \left( { - \zeta \sqrt {\frac{{a_{0} p_{0} s}}{{s + a_{1} }}} } \right). $$

Equation (25) is transformed using the inverse Laplace transform to present isothermal temperature solutions in the time domain, which yields.

26 $$ \theta_{iso} \left( {\zeta ,\tau } \right) = g_{iso} \left( {\zeta ,\tau ,a_{0} ,p_{0} ,a_{1} } \right) = 1 + \frac{{2a_{0} p_{0} }}{\pi }\int\limits_{0}^{\infty } {\frac{{\sin \left( {\zeta x} \right)}}{{x\left( {a_{0} p_{0} + x^{2} } \right)}}} \exp \left( { - \frac{{a_{1} x^{2} \zeta \tau }}{{a_{0} p_{0} + x^{2} }}} \right)dx. $$

For ramping and isothermal wall temperatures, Eqs. (21) and (26) shows the exact solution of Eq. (13), respectively.

### Solution of concentration field

The concentration equation given in Eq. (14) can be solved in the Laplace domain as follows by using the same method as in the temperature field using the Laplace transform technique:

27 $$ \frac{{d\overline{\psi }\left( {\zeta ,s} \right)}}{{d\zeta^{2} }} - \left( {\frac{{sb_{2} + b_{3} }}{{s + a_{1} }}} \right)\overline{\psi }\left( {\zeta ,s} \right) = 0, $$

upon solving the above equation, we obtain:

28 $$ \overline{\psi }\left( {\zeta ,s} \right) = \left( {\frac{1 - \exp ( - s)}{{s^{2} }}} \right)\exp \left( { - \zeta \sqrt {\frac{{sb_{2} + b_{3} }}{{s + a_{1} }}} } \right), $$

where $$b_{2} = \left( {a_{0} b_{0} - \gamma } \right)$$ and $$b_{3} = \gamma a_{1}$$.

The Ramped wall concentration solutions are presented as follows:

29 $$ \overline{\psi }\left( {\zeta ,s} \right) = \overline{\psi }_{Ramp} \left( {\zeta ,s} \right) - \exp \left( { - s} \right)\overline{\psi }_{Ramp} \left( {\zeta ,s} \right), $$

where

30 $$ \overline{\psi }_{Ramp} \left( {\zeta ,s} \right) = \overline{f}_{Ramp} (\zeta ,s,a_{1,} b_{2} ,b_{3} ) = \frac{1}{{s^{2} }}\exp \left( { - \zeta \sqrt {\tfrac{{sb_{2} + b_{3} }}{{s + a_{1} }}} } \right). $$

By inverting the Laplace transform, we obtain:

31 $$ \psi \left( {\zeta ,\tau } \right) = \psi_{Ramp} \left( {\zeta ,\tau } \right) - \psi_{Ramp} \left( {\zeta ,\tau - 1} \right)H(\tau - 1) $$

The term $\psi_{Ramp} \left( {\zeta ,\tau } \right)$ is obtained as:

32 $$ \psi_{Ramp} (\zeta ,\tau ) = \int\limits_{0}^{1} {f_{Ramp} (\zeta ,\tau ,a_{1} ,b_{2} } ,b_{3} )d\tau , $$

where

33 $$ f_{Ramp} \left( {\zeta ,\tau ;a_{1} ,b_{2} ,b_{3} } \right) = 1 + \frac{{2b_{2} }}{\pi }\int\limits_{0}^{\infty } {\frac{\sin (\zeta x)}{{x(b_{2} + x^{2} )}}} \exp \left( { - \frac{{b_{1} x^{2} \zeta \tau }}{{b_{2} + x^{2} }}} \right)dx. $$

For ramped wall boundary conditions, the precise solutions of the concentration equation are represented by Eq. (31), for $0 < t < 1.$ To determine isothermal wall concentration, Eq. (14) is again solved for isothermal wall concentration, which gives:

34 $$ \overline{\psi }_{iso} \left( {\zeta ,s} \right) = \frac{1}{s}\exp \left( { - \zeta \sqrt {\frac{{sb_{2} + b_{3} }}{{s + a_{1} }}} } \right), $$

such that

35 $$ \overline{\psi }_{iso} \left( {\zeta ,s} \right) = \overline{g}_{iso} \left( {\zeta ,s,a_{1} ,b_{2} ,b_{1} } \right) = \frac{1}{s}\exp \left( { - \zeta \sqrt {\frac{{sb_{2} + b_{3} }}{{s + a_{1} }}} } \right), $$

Equation (34) produces the following results when the inverse Laplace transform is applied for isothermal wall concentration solutions in the time domain are:

36 $$ \psi_{iso} \left( {\zeta ,\tau } \right) = g_{iso} (\zeta ,\tau ,a_{1} ,b_{2} ,b_{1} ) = 1 + \frac{{2b_{2} }}{\pi }\int\limits_{0}^{\infty } {\frac{\sin (\zeta x)}{{x(b_{2} + x^{2} )}}} \exp \left( { - \frac{{\zeta b_{1} x^{2} \tau }}{{b_{2} + x^{2} }}} \right)dx $$

For ramping and isothermal concentration, Eqs. (31) and (36) shows the exact solutions of Eq. (14) respectively.

### Solution for velocity field

Using Eq. (11) and Eq. 12 is transformed using the Laplace teqnique, the results are follows.:

37 $$ \chi_{0} \left( {\frac{{s\overline{u}\left( {\zeta ,s} \right)}}{(1 - \alpha )s + \alpha } + \beta \overline{u}\left( {\zeta ,s} \right)} \right) = \chi_{1} \frac{{d^{2} \overline{u}\left( {\zeta ,s} \right)}}{{d\zeta^{2} }} - \chi_{2} M\overline{u}\left( {\zeta ,s} \right) + \chi_{3} Gr\overline{\theta }\left( {\zeta ,s} \right) + \chi_{4} Gm\overline{\psi }\left( {\zeta ,s} \right), $$

With more simplification and the incorporation of Eqs. (18) and (28) we obtain:

38 $$ \begin{gathered} \frac{{d^{2} \overline{u}\left( {\zeta ,s} \right)}}{{d\zeta^{2} }} - \left( {\frac{{sz_{1} + z_{2} }}{{s + a_{1} }}} \right)\overline{u}\left( {\zeta ,s} \right) = - Gr_{0} \left( {\frac{1 - \exp ( - s)}{{s^{2} }}} \right)\exp \left( { - \zeta \sqrt {\frac{{sa_{0} p_{0} }}{{s + a_{1} }}} } \right) - \hfill \\ Gm_{0} \left( {\frac{1 - \exp ( - s)}{{s^{2} }}} \right)\exp \left( { - \zeta \sqrt {\frac{{sb_{2} + b_{3} }}{{s + a_{1} }}} } \right), \hfill \\ \end{gathered} $$

along with the transformed boundary conditions are:

$$\overline{u}\left( {\zeta ,s} \right) = \frac{s}{{s^{2} + \omega^{2} }}$$ and $$\overline{u}(\infty ,s) = 0$$,

where $$z_{1} = \frac{1}{{\phi_{1} }}(\phi_{2} a_{0} + \phi_{5} \beta + \phi_{2} M)$$ ,$$z_{2} = \frac{1}{{\phi_{1} }}(a_{1} \phi_{2} M + a_{1} \phi_{5} \beta )$$ , $$Gr_{0} = \frac{{\phi_{3} }}{{\phi_{1} }}Gr$$, $$\,Gm_{0} = \frac{{\phi_{4} }}{{\phi_{1} }}Gm$$

In the Laplace domain, Eq. (38) has the following solution:

39 $$ \begin{gathered} \overline{u}\left( {\zeta ,s} \right) = \frac{s}{{s^{2} + \omega^{2} }}\exp \left( { - \zeta \sqrt {\frac{{sz_{1} + z_{2} }}{{s + a_{1} }}} } \right) + \left( {\frac{{Gr_{0} (s + a_{1} )}}{{a_{10} s - z_{2} }}} \right)\left( {\frac{1 - \exp ( - s)}{{s^{2} }}} \right) \hfill \\ \,\,\,\,\,\,\,\,\,\,\,\,\,\,\,\,\,\left( {\exp \left( { - \zeta \sqrt {\frac{{sz_{1} + z_{2} }}{{s + a_{1} }}} } \right) - \exp \left( { - \zeta \sqrt {\frac{{sa_{0} p_{0} }}{{s + a_{1} }}} } \right)} \right) \hfill \\ \,\,\,\,\,\,\,\,\,\,\,\,\,\,\,\,\,\,\, + \left( {\frac{{Gm(s + a_{1} )}}{{a_{2} s - z_{2} }}} \right)\left( {\frac{1 - \exp ( - s)}{{s^{2} }}} \right) \hfill \\ \,\,\,\,\,\,\,\,\,\,\,\,\,\,\,\,\,\,\left( {\exp \left( { - \zeta \sqrt {\frac{{sz_{1} + z_{2} }}{{s + a_{1} }}} } \right) - \exp \left( { - \zeta \sqrt {\frac{{sb_{2} + b_{3} }}{{s + a_{1} }}} } \right)} \right)\,,\,\,\,\, \hfill \\ \end{gathered} $$

where $$a_{0} p_{0} - a_{1} = a_{10}$$

The more appropriate form of Eq. (39) is:

40 $$ \overline{u}\left( {\zeta ,s} \right) = \left( \begin{gathered} \overline{u}_{c} \left( {\zeta ,s} \right) + \overline{u}_{1} \left( {\zeta ,s} \right)\left( {\overline{u}_{{2\left( {Ramp} \right)}} \left( {\zeta ,s} \right) - e^{ - s} \overline{u}_{{2\left( {Ramp} \right)}} \left( {\zeta ,s} \right)} \right) \hfill \\ + \overline{u}_{3} \left( {\zeta ,s} \right)\left( {\overline{u}_{{2\left( {Ramp} \right)}} \left( {\zeta ,s} \right) - e^{ - s} \overline{u}_{{2\left( {Ramp} \right)}} \left( {\zeta ,s} \right)} \right) \hfill \\ - \overline{u}_{1} \left( {\zeta ,s} \right)\overline{\theta }\left( {\zeta ,s} \right) - \overline{u}_{3} \left( {\zeta ,s} \right)\overline{\psi }\left( {\zeta ,s} \right) \hfill \\ \end{gathered} \right) $$

where,

41 $$ \overline{u}_{c} \left( {\zeta ,s} \right) = \frac{s}{{s^{2} + \omega^{2} }}\exp \left( { - \zeta \sqrt {\frac{{sz_{1} + z_{2} }}{{s + a_{1} }}} } \right), $$

42 $$ \overline{\psi }_{1} (\eta ,s) = \left( {\frac{{Gr_{0} (s + b_{1} )}}{{a_{3} s - a_{2} }}} \right), $$

43 $$ \overline{u}_{3} (\zeta ,s) = \left( {\frac{{Gm_{0} (s + a_{1} )}}{{a_{2} s - z_{2} }}} \right), $$

44 $$ \overline{u}_{{2\left( {Ramp} \right)}} (\zeta ,s) = \exp \left( { - \zeta \sqrt {\frac{{sz_{1} + z_{2} }}{{s + a_{1} }}} } \right). $$

Equation (40) takes the following form when the Laplace transform is inverted:

45 $$ u\left( {\zeta ,\tau } \right) = \left( \begin{gathered} u_{c} \left( {\zeta ,\tau } \right) + u_{1} \left( {\zeta ,\tau } \right)*\left( {u_{{2\left( {Ramp} \right)}} \left( {\zeta ,\tau } \right) - H\left( {\tau - 1} \right)u_{{2\left( {Ramp} \right)}} \left( {\zeta ,\tau - 1} \right)} \right) \hfill \\ + u_{3} \left( {\zeta ,\tau } \right)*\left( {u_{{2\left( {Ramp} \right)}} \left( {\zeta ,\tau } \right) - H\left( {\tau - 1} \right)u_{{2\left( {Ramp} \right)}} \left( {\zeta ,\tau - 1} \right)} \right) \hfill \\ - u_{1} \left( {\zeta ,\tau } \right)*\theta \left( {\zeta ,\tau } \right) - u_{3} \left( {\zeta ,\tau } \right)*\psi \left( {\zeta ,\tau } \right) \hfill \\ \end{gathered} \right), $$

46 $$ u_{c} \left( {\zeta ,\tau } \right) = e^{{ - \zeta \sqrt {a_{1} } }} - \int\limits_{0}^{\infty } {\int\limits_{0}^{\tau } {\cos \left( {\omega (\tau - t)} \right)} } \frac{1}{u}\frac{{\zeta \sqrt {a_{4} } }}{{2\sqrt {\pi t} }}e^{{\frac{{\zeta^{2} }}{4u} - \gamma_{1} t - \lambda_{1} uI_{1} }} (2\sqrt {a_{4} ut} )dtdu $$

47 $$ u_{1} (\zeta ,\tau ) = \left( {a_{4} e^{{\frac{{z_{2} }}{{z_{3} }}}} + \frac{1}{{z_{3} }}\delta (\tau )} \right) $$

48 $$ u_{3} (\zeta ,\tau ) = \left( {a_{7} e^{{\frac{{z_{1} }}{{z_{2} }}}} + \frac{1}{{z_{1} }}\delta (\tau )} \right) $$

49 $$ u_{2(Ramp)} \left( {\zeta ,\tau } \right) = e^{{\zeta \sqrt {a_{1} } }} - \int\limits_{0}^{\infty } {\int\limits_{0}^{\tau } {(\tau - t)\frac{1}{u}} } \frac{{\zeta \sqrt {a_{4} } }}{{\sqrt {\pi t} }}e^{{\frac{{\zeta^{2} }}{4u} - \gamma_{1} t - \lambda_{1} uI_{1} }} (2\sqrt {a_{4} ut} )dtdu $$

here

$$ \begin{gathered} a_{4} = z_{2} - z_{1} a_{1} a_{5} = Gr_{0} \left( {\frac{{z_{2} + a_{3} a_{1} }}{{a_{3}^{2} }}} \right), \hfill \\ a_{7} = z_{2} - a_{2} a_{5} = Gm_{0} \left( {\frac{{z_{2} + a_{6} a_{1} }}{{a_{6}^{2} }}} \right), \hfill \\ \end{gathered} $$

where the convolution product is represented by *. It's important to note that Eq. (45) refers to the ramping wall boundary conditions for the velocity field solutions.

Now Eq. (12) is re-solved for isothermal boundary conditions, which yields us the final solution in the form:

50 $$ u_{iso} \left( {\zeta ,\tau } \right) = \left( \begin{gathered} u_{c} \left( {\zeta ,\tau } \right) + u_{1} \left( {\eta ,\tau } \right)*\left( {u_{{2\left( {iso} \right)}} \left( {\zeta ,\tau } \right) - H\left( {\tau - 1} \right)u_{{2\left( {iso} \right)}} \left( {\zeta ,\tau - 1} \right)} \right) \hfill \\ + u_{3} \left( {\zeta ,\tau } \right)*\left( {u_{{2\left( {iso} \right)}} \left( {\zeta ,\tau } \right) - H\left( {\tau - 1} \right)u_{{2\left( {iso} \right)}} \left( {\zeta ,\tau - 1} \right)} \right) \hfill \\ - u_{1} \left( {\zeta ,\tau } \right)*\theta_{iso} \left( {\zeta ,\tau } \right) - u_{3} \left( {\zeta ,\tau } \right)*\psi_{iso} \left( {\zeta ,\tau } \right) \hfill \\ \end{gathered} \right), $$

where

51 $$ u_{4(iso)} (\zeta ,\tau ) = e^{{ - \zeta \sqrt {z_{1} } }} - \int\limits_{0}^{\infty } {\int\limits_{0}^{\infty } {\frac{1}{\psi }} } \frac{{\zeta \sqrt {a_{4} } }}{{2\sqrt {\pi \tau } }}e^{{\frac{\eta }{4\psi } - a_{1} \tau - z_{1} uI_{1} }} (2\sqrt {a_{4} \psi \tau } )d\tau du, $$

and $u_{c} \left( {\zeta ,\tau } \right),\,\,\,u_{1} \left( {\zeta ,\tau } \right),\,\theta_{iso} \left( {\zeta ,\tau } \right)\,\,{\text{and}}\,\,\psi_{iso} \left( {\zeta ,\tau } \right)$ are previously defined.

## Limiting cases

Some limiting cases are derived to validate the current work from our general solutions as following:

*Case* 1: In the absence of $Gm = 0$ and $Nr = 0$ our obtained results are relatively the same as those of Saqib et al*.*^53^

52 $$ \begin{gathered} \chi_{0} \left( {{}^{CF}D_{\tau }^{\alpha } u\left( {\zeta ,\tau } \right) + \beta u\left( {\zeta ,\tau } \right)} \right) = \chi_{1} \frac{{\partial^{2} u\left( {\zeta ,\tau } \right)}}{{\partial \zeta^{2} }} - \chi_{2} Mu\left( {\zeta ,\tau } \right)  \hfill \\ +\chi_{3} Gr\theta \left( {\zeta ,\tau } \right). \hfill \\ \end{gathered} $$

*Case* 2: In the absence of $q_{r} = 0$ and $Nr = 0$ our obtained results are similar to the Hasin et al.^30^*.*

53 $$ \begin{gathered} \chi_{0} \left( {{}^{CF}D_{\tau }^{\alpha } u\left( {\zeta ,\tau } \right) + \beta u\left( {\zeta ,\tau } \right)} \right) = \chi_{1} \frac{{\partial^{2} u\left( {\zeta ,\tau } \right)}}{{\partial \zeta^{2} }} - \chi_{2} Mu\left( {\zeta ,\tau } \right) \hfill \\ +\chi_{3} Gr\theta \left( {\zeta ,\tau } \right) + \chi_{4} Gm\psi \left( {\zeta ,\tau } \right), \hfill \\ \end{gathered} $$

## Nusselt number, sherwood number, and skin friction

### Nusselt number

The Nusselt number is expressed mathematically as

54 $$ Nu = - \frac{{k_{nf} }}{{k_{f} }}\left. {\frac{\partial \theta }{{\partial \zeta }}} \right|_{\zeta = 0} $$

### Sherwood number

The Sherwood number is expressed mathematically as:

55 $$ S_{h} = - D_{nf} \left. {\frac{\partial \psi }{{\partial \zeta }}} \right|_{\zeta = 0} $$

### Skin friction

The skin friction is expressed mathematically as:

56 $$ Sf\left( {\zeta ,\tau } \right) = \frac{1}{{\left( {1 - \phi } \right)^{2.5} }}\left. {\frac{\partial u}{{\partial \zeta }}} \right|_{\zeta = 0} $$

## Graphical results and discussion

**Figure 2:**
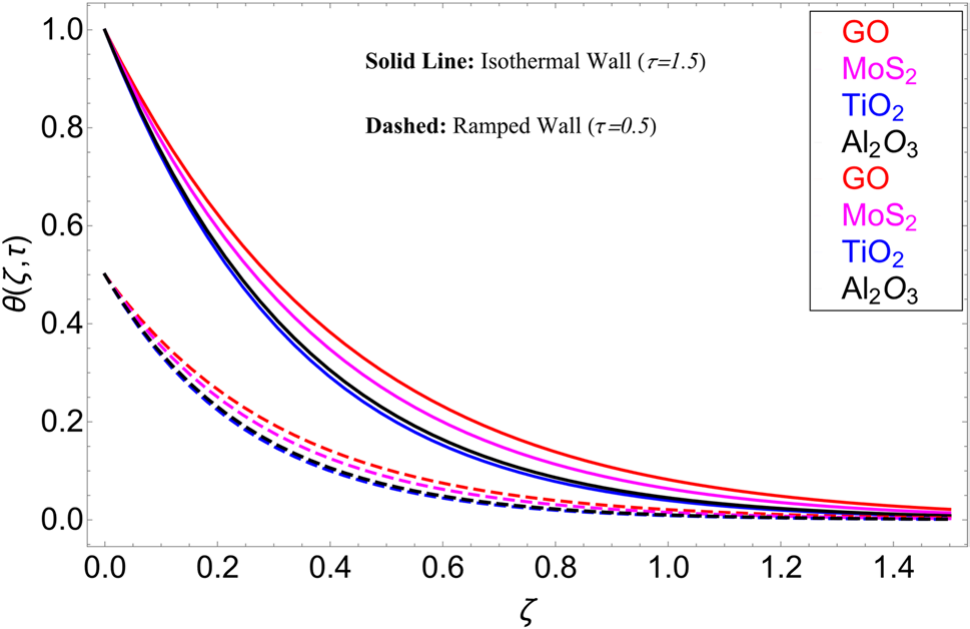
Temperature distribution for different types of nanoparticles.

**Figure 3:**
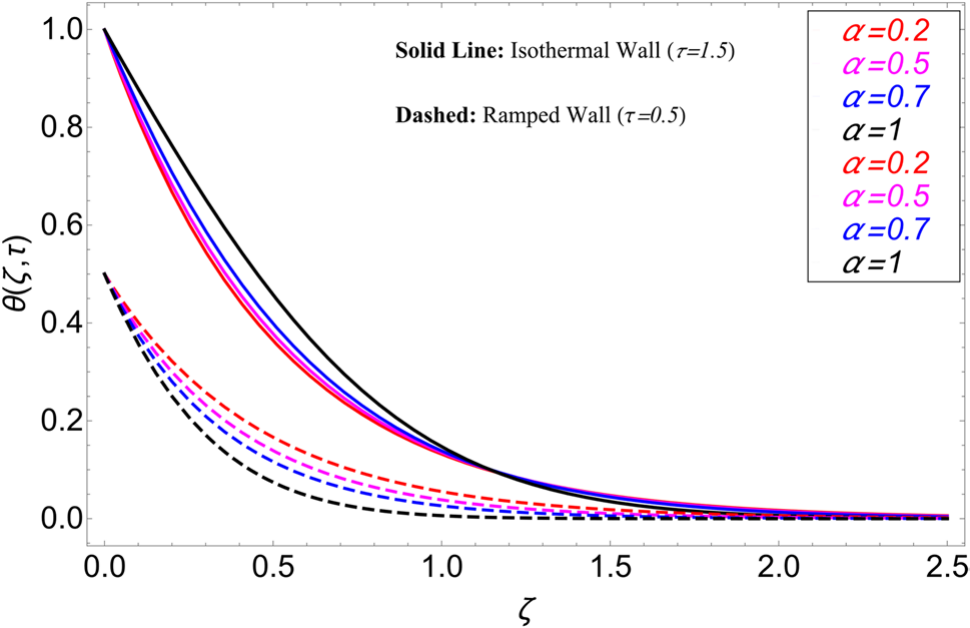
Temperature distribution for different values of $\alpha$.

**Figure 4:**
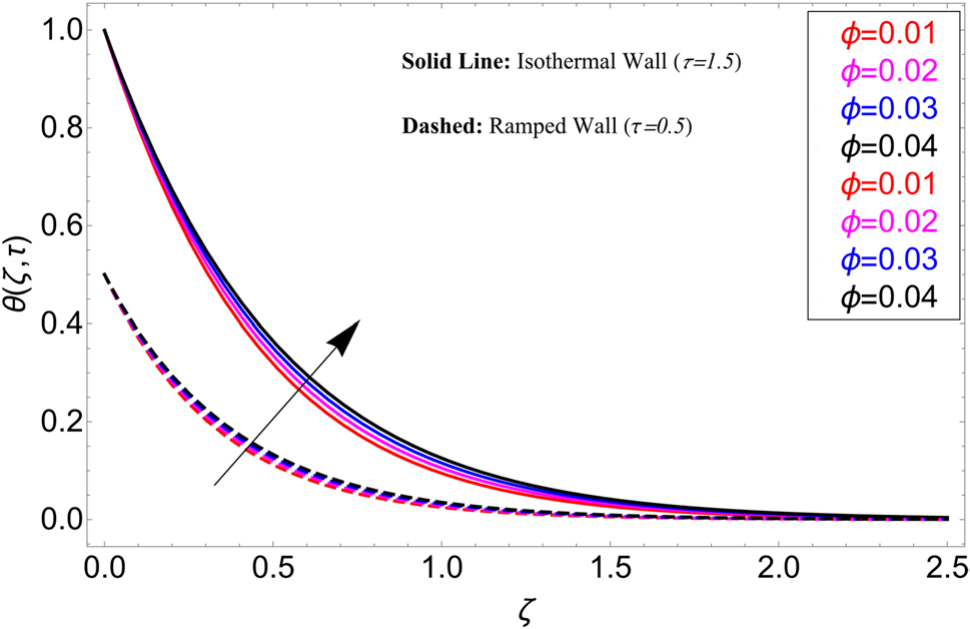
Temperature distribution for different values of $\phi$.

**Figure 5:**
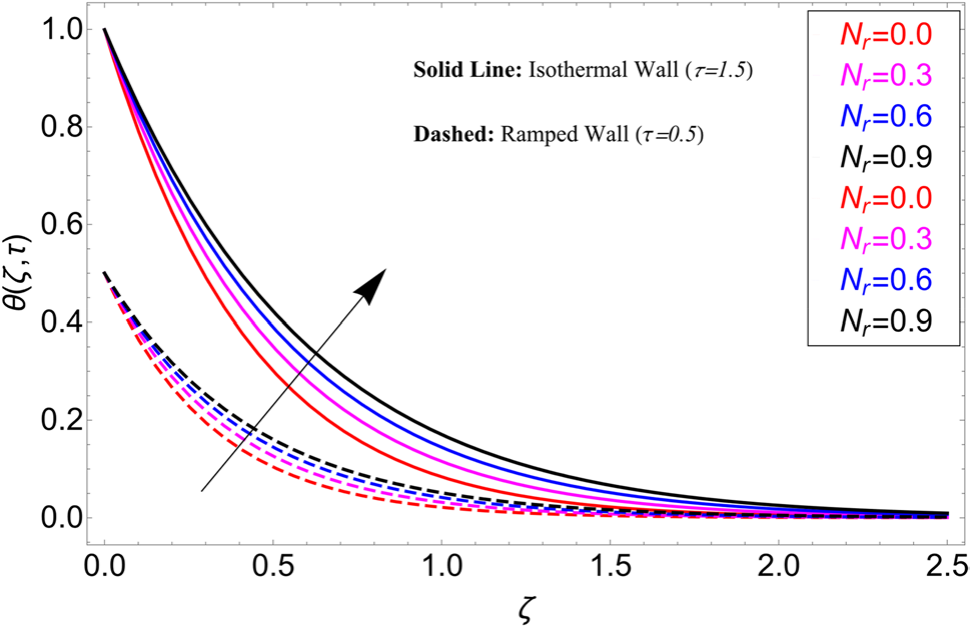
Temperature distribution for different values of $N_{r}$.

**Figure 6:**
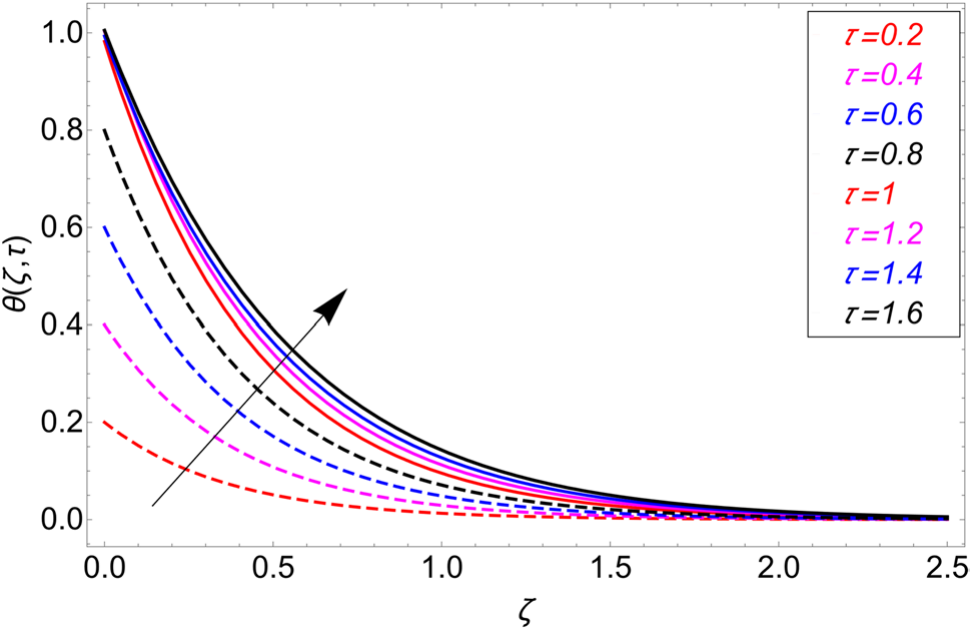
Temperature distribution for different values of $\tau$.

**Figure 7:**
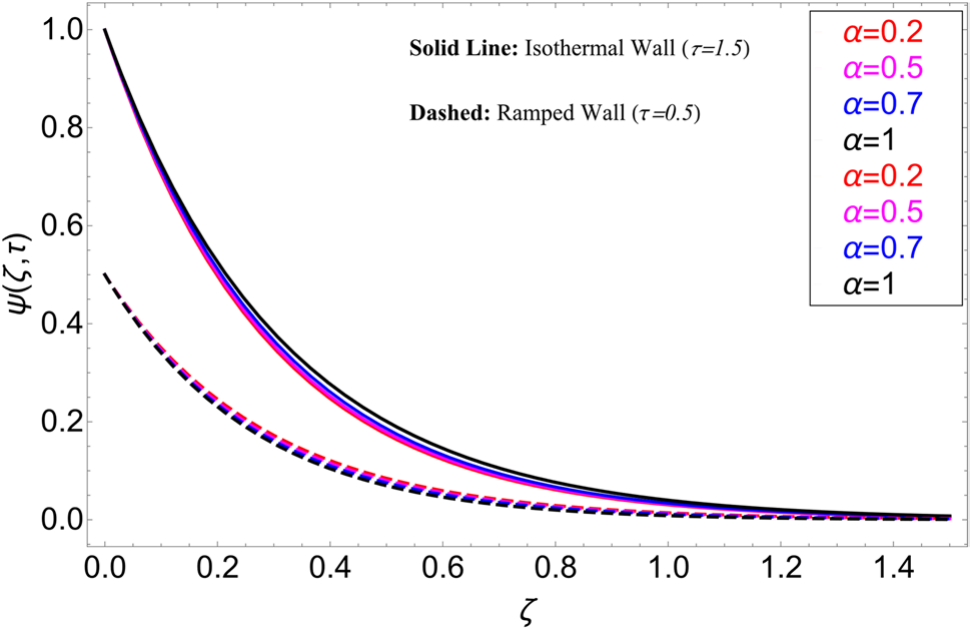
Concentration distribution for different values of $\alpha$.

**Figure 8:**
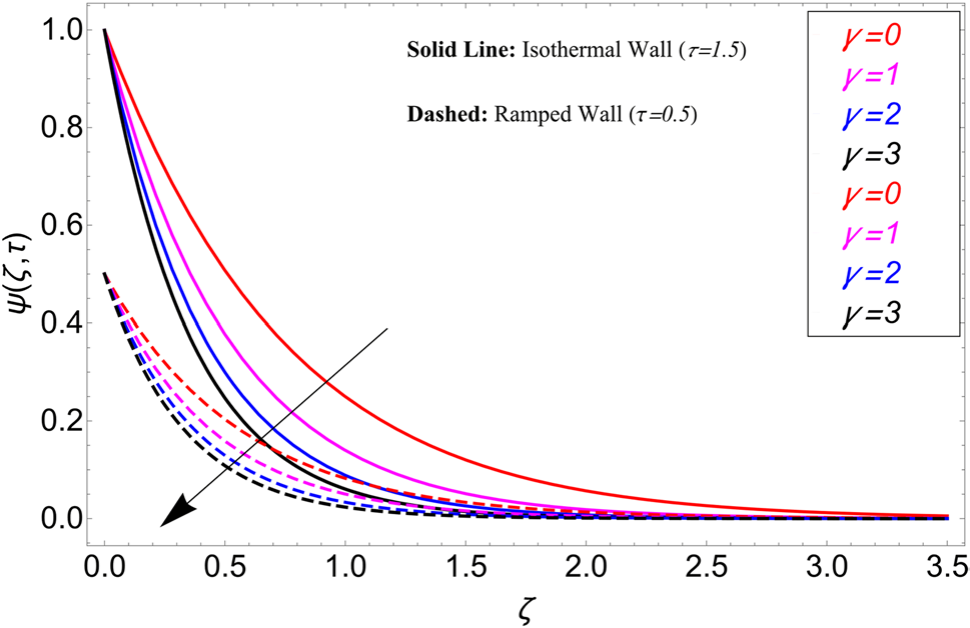
Concentration distribution for different values of $\gamma$.

**Figure 9:**
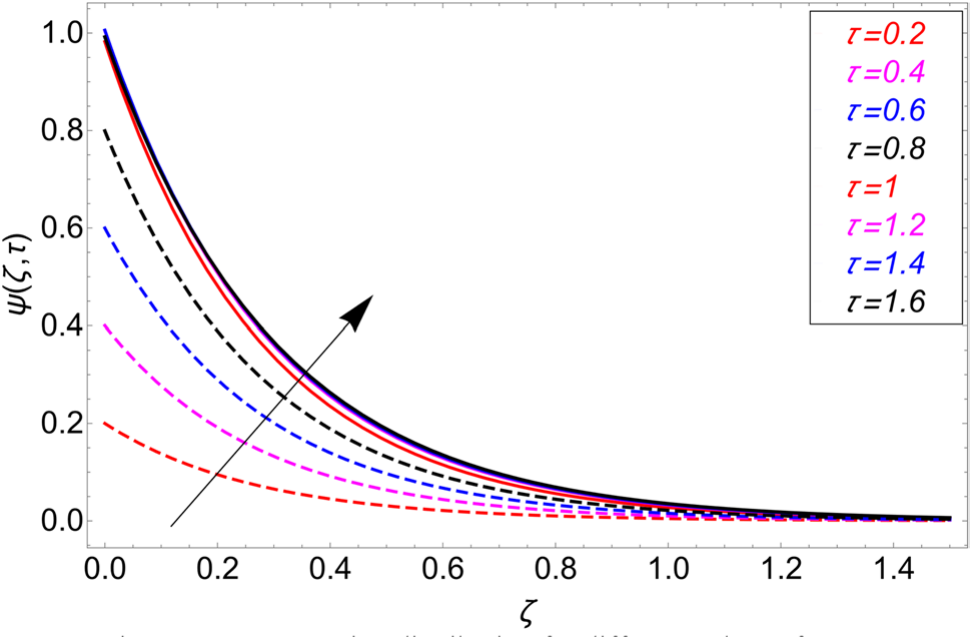
Concentration distribution for different values of $\tau$.

**Figure 10:**
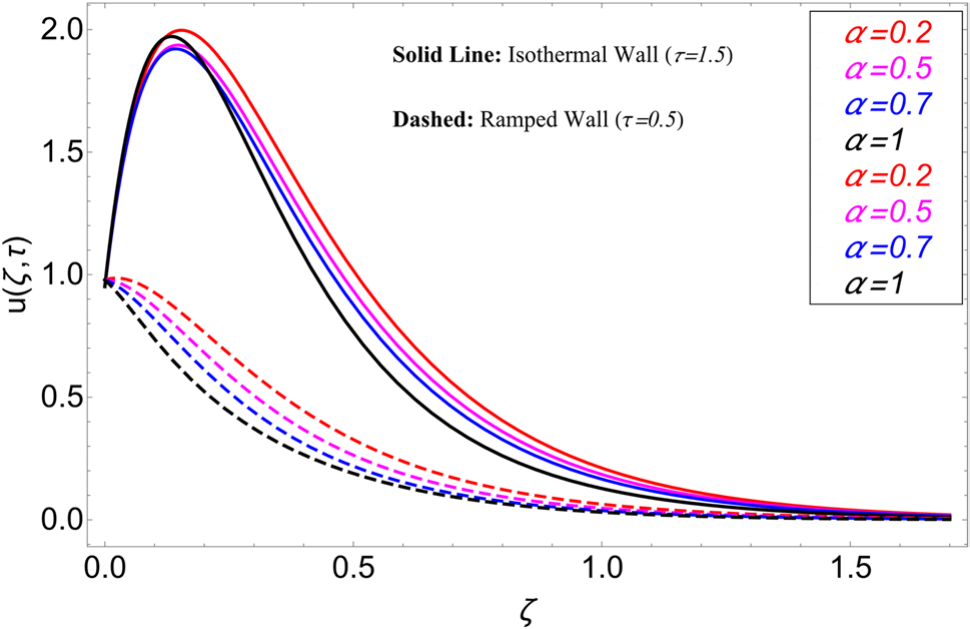
Velocity distribution for different values of $\alpha$.

**Figure 11:**
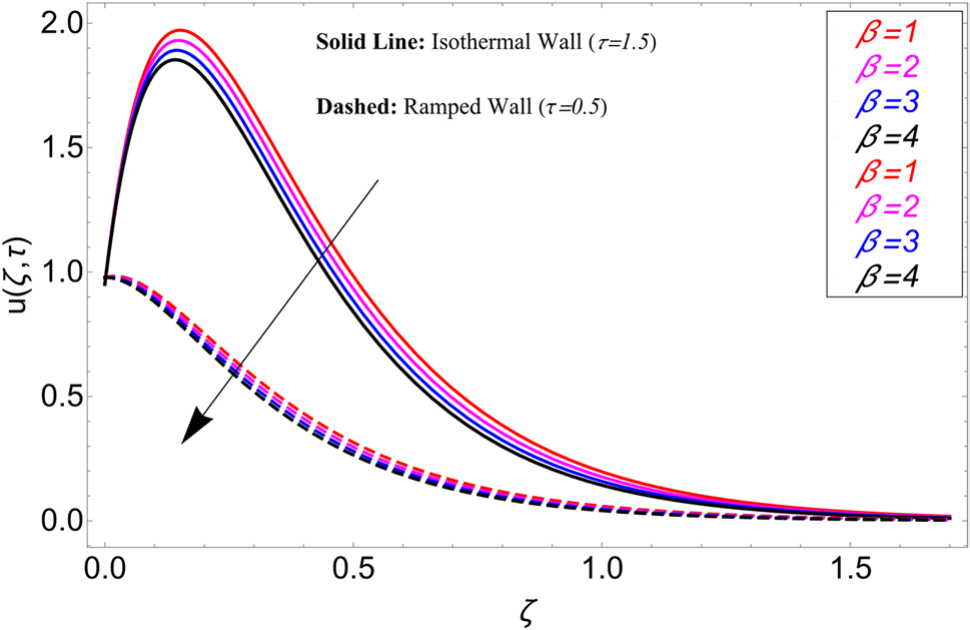
Velocity distribution for different values of $\beta$.

**Figure 12:**
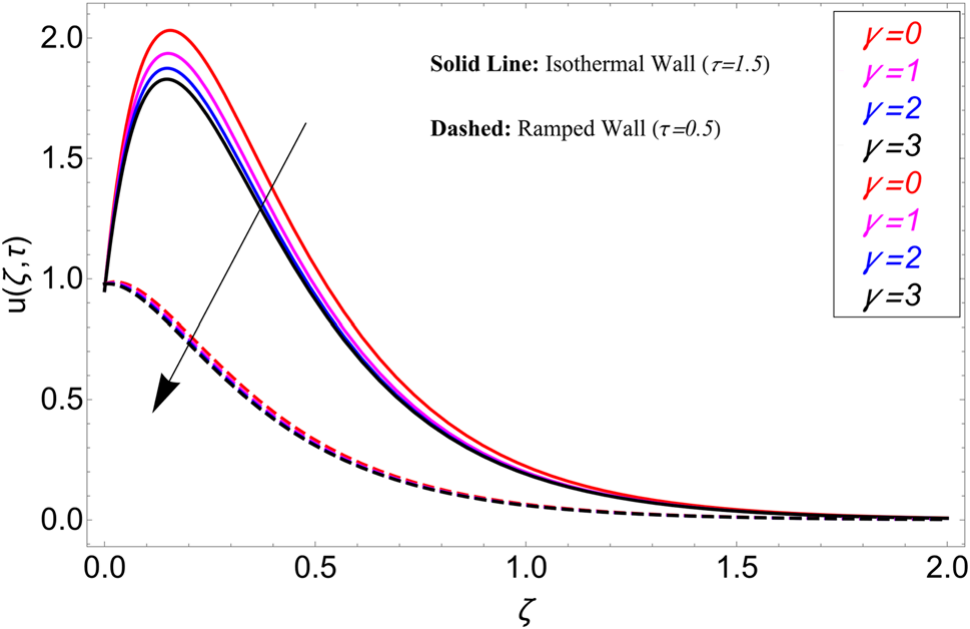
Velocity distribution for different values of $\gamma$.

**Figure 13:**
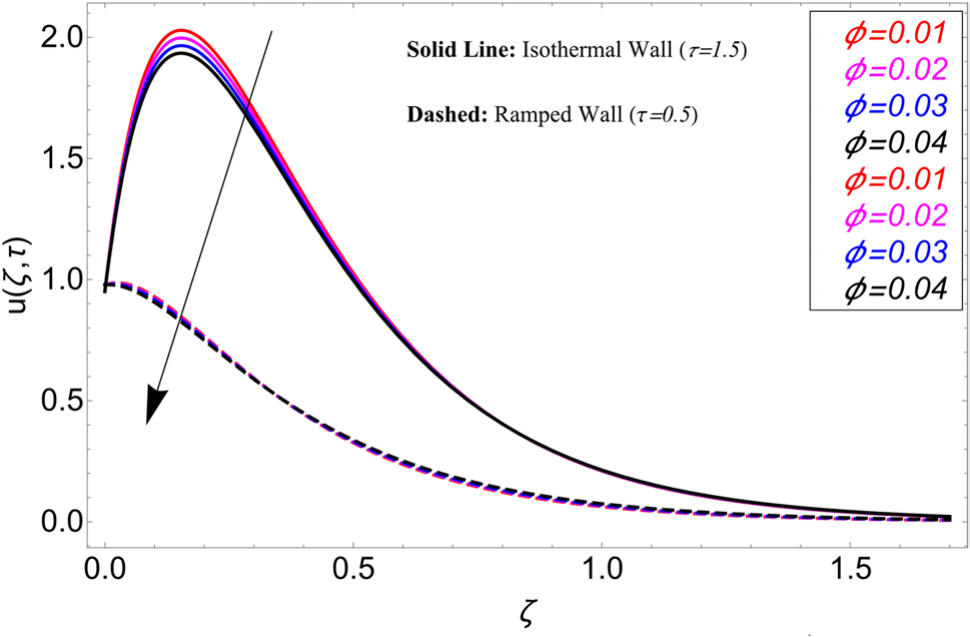
Velocity distribution for different values of $\phi .$

**Figure 14:**
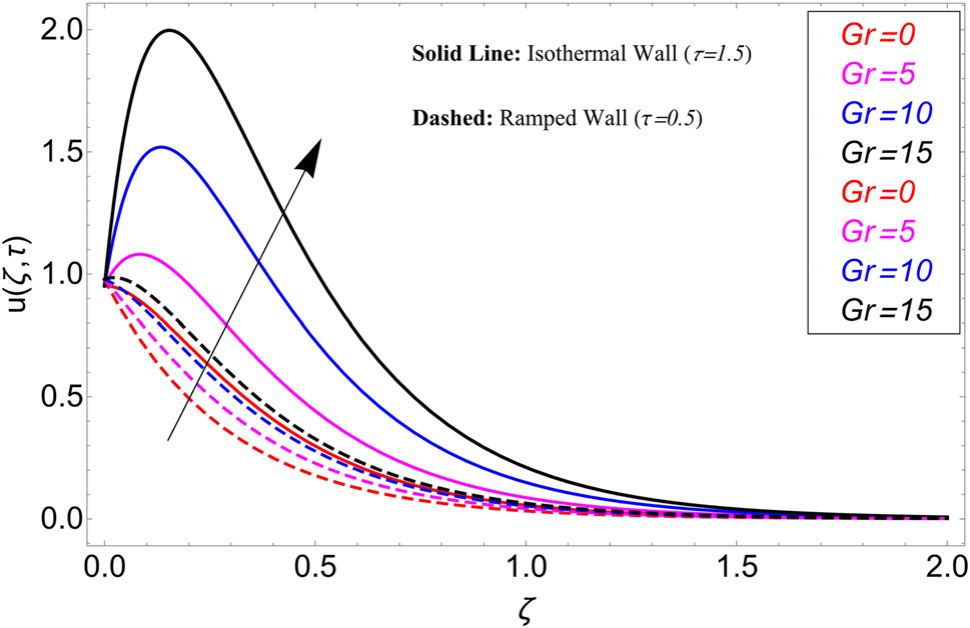
Velocity distribution for different values of *Gr*.

**Figure 15:**
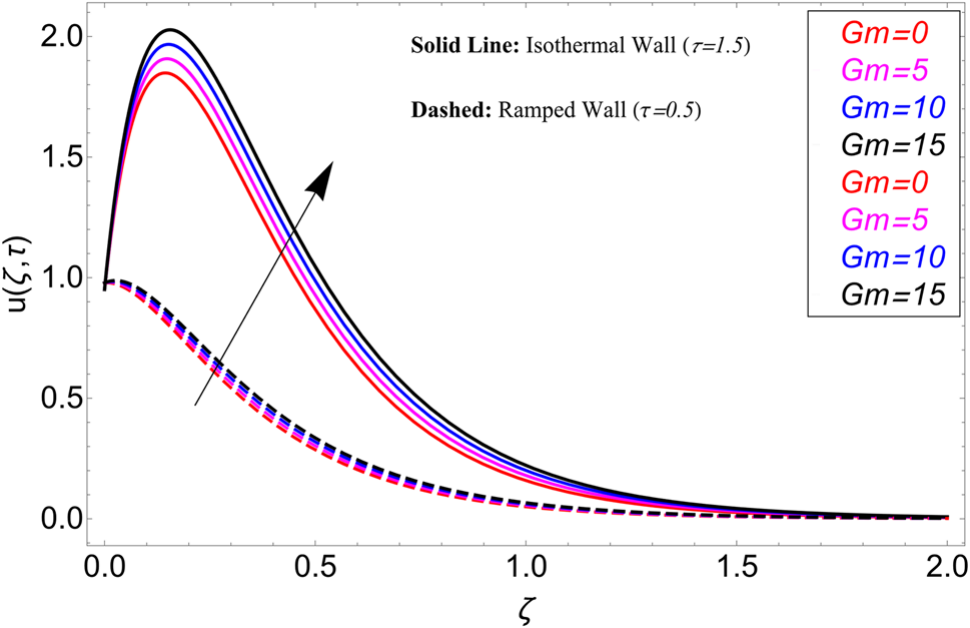
Velocity distribution for different values of *Gm*.

**Figure 16:**
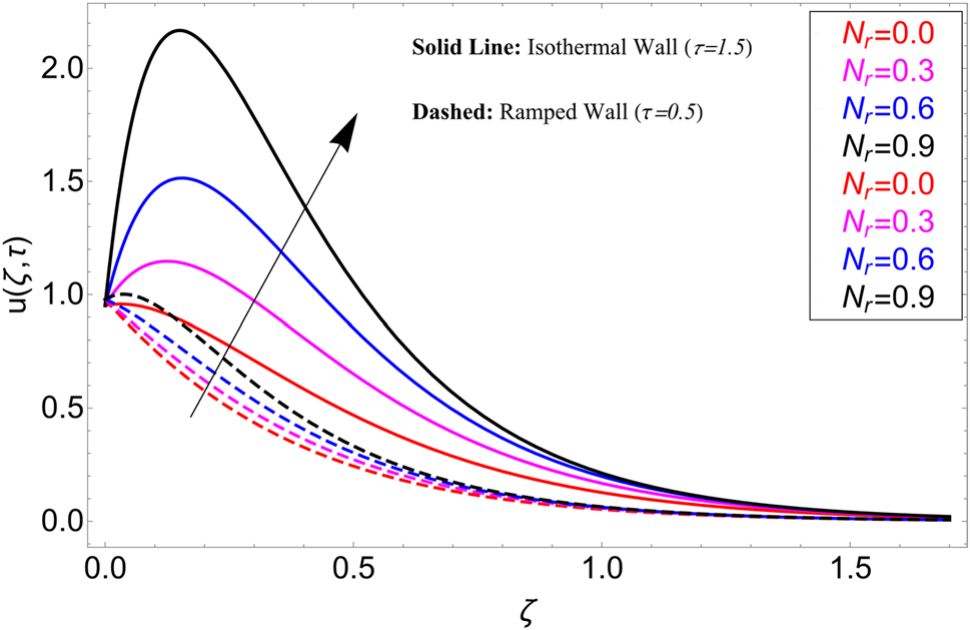
Velocity distribution for different values of $N_{r}$.

**Figure 17:**
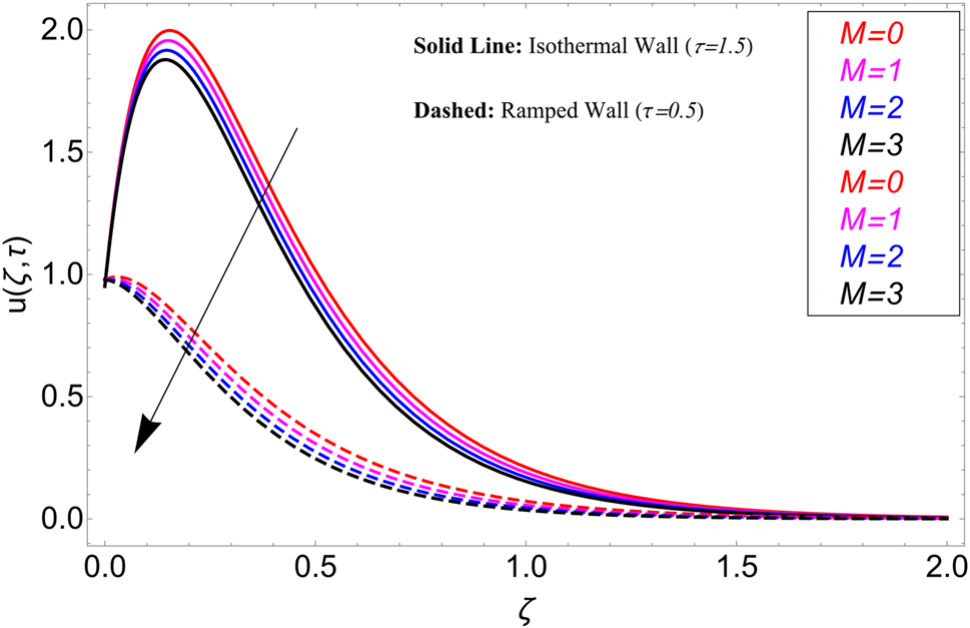
Velocity distribution for different values of $M$.

In the current study, a nanofluid's magnetohydrodynamic flow is examined of the Brinkman type which flowing across a vertical plate. We also look at how temperature and concentration are affected by ramping and isothermal boundary conditions. The combined effect of chemical reaction thermal radiation is also investigated. The Caputo-Fabrizio fractional derivative is used to expand the classical model. Additionally, vegetable oil is chosen as the base fluid, while four different types of nanoparticles (GO, M_O_S_2_, TiO_2,_ and Al_2_O_3_) are mixed in regular vegetable oil to improve its thermal qualities. Exact results are gained applying the Laplace transform approach. Closed-form solutions are computed for temperature, concentration, and velocity fields. Figure 1 shows how the current work is physically represented. Figures 2, 3, 4, 5, 6, 7, 8, 9, 10, 11, 12, 13, 14, 15, 16 and 17 provide a graphic representation of the temperature, velocity, and concentration distributions. To see the differences clearly, $\tau = 0.5$ and $\tau = 1.5$ are used for ramped and isothermal wall conditions, respectively. Table 1 lists the base fluid, vegetable oil, and considered nanoparticles' thermophysical characteristics. Skin friction, Sherwood number, and Nusselt number are computed and tabulated and are given in Tables 2, 3 and 4.

This study is carried out for the comparison among different types of nanoparticles (GO, M_O_S_2_, TiO_2,_ and Al_2_O_3_). These nanoparticles are considered equally dispersed in the based fluid (vegetable oil), which is considered a cutting fluid. Figure 2 depicts the results of these various types of nanoparticles on the temperature profile. From this figure, it can be observed that the temperature profile is higher for GO which is followed by M_O_S_2_, TiO_2,_ and Al_2_O_3_. The effect of $\alpha$ on the temperature profile for both ramping and isothermal wall boundary conditions is clearly shown in Fig. 3. Comparatively, fractional models are more inclusive than classical models. For various values of, fractional models provide us with multiple solutions, which means that experimentalists can choose which solution best fits their results. Additionally, the present fractional model’s solution is reduced to classical order by taking $\alpha \to 1$. The influence $\phi$ on the temperature profile is cleared in Fig. 4. By increasing $\phi$, the temperature field increases. As the amount of nanoparticles increases, the heat transfer rate of the base fluid increases, which increases the temperature field. As seen in Fig. 5, increasing the radiation parameter Nr, moreover the temperature profile rises. This effect is clear because the rate of radiation released from the fluid is precisely proportional to a temperature that raises the temperature field. The impact of time $\tau$ on the temperature profile is depicted in Fig. 6. It has been discovered that both circumstances (ramped and isothermal boundary conditions), $\tau$ increases the temperature of the fluids. Additionally, it is discovered that under ramping boundary conditions i.e., $0 < \tau < 1$, whereas in case of isothermal boundary conditions, the temperature at the boundary fluctuates ($\tau \ge 1$), and The temperature reaches its highest point and doesn't change for isothermal case.

The influence $\alpha$ for the concentration profile is cleared from Fig. 7. It has been examined that taking the fractional model into consideration provides with several concentration profile curves. It also provides us with options for a concentration profile in the conventional sense by taking $\alpha = 1$. Figure 8 illustrates how the chemical reaction parameter $\gamma$ affects the concentration profile. It is evident from this graph that raising the chemical reaction parameter's value causes the fluid concentration to decrease more quickly. The fluid reacts, which causes the fluid flow rate to rise and lower the concentration profile. Figure 9 shows the effect of $\tau$ on the concentration profile. It has been observed that it extends the concentration field in both circumstances. Additionally, it is noted that, in the event of isothermal boundary circumstances i.e.,$\tau \ge 1$ the concentration achieves its maximum and does not vary, in contrast to ramping boundary conditions, i.e., $0 < \tau < 1$ which cause the border concentration to change.

The influence $\alpha$ on the velocity field is cleared from Fig. 10 for ramping and isothermal wall boundary cases. By comparison of fractional models to the classical models It has been noted that the fractional models are more realistic and general, to excellently match their findings to theoretical findings, It is impossible under the classical model for $\alpha = 1$. Additionally, the present fractional model’s solution is also reduceable to classical order by taking $\alpha \to 1$. As seen in Fig. 11, the effect of $\beta$ on the velocity profile. The relationship between the drag force and $\beta$ is directly proportional. The greater the value of $\beta$, The velocity field is reduced as the drag forces get stronger. Figure 12 depicts how the velocity profile is affected by the chemical reaction parameter $\gamma$. The velocity of the nanofluid decreases as the chemical reaction parameter $\gamma$ is increases. Chemical processes change the fluid's behavior and cause it to become denser, hence the denser fluid's velocity is the lowest. Figure 13 shows how the volume fraction parameter $\phi$ affects the velocity of nanofluids. For both ramping and isothermal boundary conditions, increasing the volume fraction parameter decreases the nanofluid velocity. This is an accurate effect because as the volume fraction grows, the number of friction forces increases, and as a result, velocity drops. The influence of Gr and Gm on the velocity field is shown in Figs. 14 and 15. In both cases, the nanofluid's velocity increases. Since increasing Gr and Gm, this velocity pattern is evident. causes the fluid motion to accelerate by increasing buoyant forces while lowering viscous forces. By increasing the radiation parameter Nr while maintaining the other values constant, the velocity profile is displayed in Fig. 16. Figure 17 illustrates the influence of *M* on the velocity profile. When M is increased, fluid velocity reduces. The science behind this states that as M grows., Lorentz forces develop, opposing the motion of the fluid and producing resistive forces on its flow which case lowers velocity of the fluid.

In numerical form, Table 2 shows the impact of various embedded characteristics on skin friction. In order to clearly illustrate the differences, the effect is given for both cases i.e., classical as well as fractional order. Similarly, The impact of volume fraction is cleared from Table 3, against the Nusselt number. As we already mentioned above that in the present analysis, we have carried out our simulations for four different types of nanoparticles (GO, MoS_2_, TiO_2,_ and Al_2_O_3_) which are used in a single based fluid vegetable oil which is considered as a cutting fluid. From the present analysis given in Table 3, the highest rate of heat transfer can be achieved by considering GO nanoparticles followed by MoS_2_, TiO_2_ and Al_2_O_3_. By dispersing 4% of nanoparticles ($\phi = 0.04$), the rate of heat transfer considering GO is observed as highest with an enhancement of 19.83% which is followed by MoS_2_ with 16.96%, TiO_2_ with 16.25%, and Al_2_O_3_ with 15.80% heat transfer enhancement. Vegetable oil is mostly used for machining and cutting purposes of heavy metals in big as well as small industries. It is also known that MoS_2_ is also used as a dry lubricant and dispersing it in the regular vegetable oil will of course enhance the lubricity along with heat transfer rate. According to the findings of this study, GO has the highest heat transfer rate, which will improve the heat transfer rate and be more efficient for the machining parts and sharp edges of the cutting tools for cutting huge metals. In the same way, the amount of mass transfer against the number of nanoparticles is given in Table 4. From the table, it is observed that decreases the mass transfer rate up to 3.74% by adding 4% of nanoparticles i.e., $\phi = 0.04$. This is due to nanoparticles' effect on nanofluid viscosity. The viscosity of the base fluid is increased by adding nanoparticles, which reduces the mass transfer rate.

**Table 1:** Vegetable oil thermophysical characteristics and different types of Nanoparticles:

Material	$\rho (Kgm^{ - 3} )$	$c_{p} (JKg^{ - 1} K^{ - 1} )$	$k(wm^{ - 1} K^{ - 1} )$	$\beta \times 10^{ - 5} (K^{ - 1} )$	$\sigma \left( {S/m} \right)$
Vegetable oil	904.3	1670	0.192	6.4X10^–4^	0.34
GO	1800	717	5000	0.284	$3.2 \times 10^{ - 4}$
MoS_2_	5.06 × 10^3^	397.21	904.4	2.8424	$2.09 \times 10^{ - 4}$
TiO_2_	425	6862	8.9538	0.9	10^–12^
Al_2_O_3_	3970	765	40	0.85	10^–10^

**Table 2:** Skin friction variation against different parameters:

*Gr*	*Gm*	*Re*	$\beta$	*T*	$\phi$	*M*	$N_{r}$	$\gamma$	$\alpha$	$C_{f}^{\alpha }$	$C_{f}^{classical}$
5	5	2	2	1	0.03	2	3	2	0.5	1.536	1.387
7	5	2	2	1	0.03	2	3	2	0.5	1.434	1.213
5	7	2	2	1	0.03	2	3	2	0.5	1.421	1.402
5	5	2.5	2	1	0.03	2	3	2	0.5	1.553	1.455
5	5	2	3	1	0.03	2	3	2	0.5	1.342	1.393
5	5	2	2	1.5	0.03	2	3	2	0.5	1.212	1.104
5	5	2	2	1	0.04	2	3	2	0.5	1.878	1.428
5	5	2	2	1	0.03	3	3	2	0.5	1.763	1.452
5	5	2	2	1	0.03	2	4	2	0.5	1.493	1.303
5	5	2	2	1	0.03	2	3	3	0.5	1.423	1.354
5	5	2	2	1	0.03	2	3	2	0.7	1.672	1.387

**Table 3:** Variation in Nusselt number for various values of $\phi$.

$\phi$	*Nu for GO*	% age Enhan-cement	*Nu for * *MoS*_*2*_	% age Enhan-cement	*Nu for TiO*_*2*_	% age Enhan-cement	*Nu for Al*_*2*_*O*_*3*_	% age Enhan-cement
0.00	3.4219	–	3.4219	–	3.4219	–	3.4219	–
0.01	3.6149	5.64%	3.5747	4.46%	3.5510	3.78%	3.5382	3.40%
0.02	3.7286	8.96%	3.7013	8.17%	3.6709	7.28%	3.6522	6.73%
0.03	3.9248	14.70%	3.8828	13.47%	3.8221	11.69%	3.7828	10.54%
0.04	4.1085	19.82%	4.0025	16.96%	3.9782	16.25%	3.9625	15.80%

**Table 4:** Variation in Sherwood number for various values of $\phi$.

$\phi$	$S_{h}$	Decrease in mass distribution
0.00	1.925	–
0.01	1.908	0.88%
0.02	1.899	1.35%
0.03	1.881	2.28%
0.04	1.853	3.74%

## Conclusion

In this work closed-form solutions for nanofluid flow of the Brinkman- type fluids over an infinite vertical plate. The fluid's temperature, concentration, thermal radiation, and chemical reaction are all examined in relation to the ramping and isothermal wall boundary conditions using the magnetic field. The four types of nanoparticles used are as: (GO, MoS_2_, TiO_2_ and Al_2_O_3_), while the base fluid is vegetable oil. The Caputo-Fabrizio fractional derivative, which has lately developed as the most popular fractional derivative, is then used to generalize the classical model. Utilizing the Laplace transform method, the coupled system's solutions are produced. A classical order system of coupled PDEs is used to model the suggested flow issue, which is then fractionalized using the Caputo-Fabrizio fractional operator. The collected results are also shown in the graphs. The main finding are as follows:

- For large values of $\alpha$, in ramped wall boundary conditions, a drop in nanofluid velocity can be noticed.
- For isothermal wall boundary conditions, however, the reverse effect is seen.
- The highest transfer rate can be achieved by considering graphene oxide *GO* nanoparticles followed by $MoS_{2}$, $TiO_{2}$ and $Al_{2} O_{3}$.
- By dispersing 4% of nanoparticles ($\phi = 0.04$), When considering GO, the maximum heat transfer rate is seen with an enhancement of 19.83% which is followed by $MoS_{2}$ with 16.96%, $TiO_{2}$ with 16.25% and $Al_{2} O_{3}$ with 15.80% heat transfer enhancements.
- As chemical reaction parameters $\gamma$ are increased, the nanofluid's velocity drops.
- There is a rise in the radiation parameter Nr, so increases the velocity profile.
- The velocity profile decreases for larger values of *M* and $\beta$.
- The current findings can be reduced to the traditional nanofluid Brinkman type model by taking $\alpha \to 1$.
- The radiation parameter Nr is being increased, so increases the velocity profile.

## Future suggestions

To solve this problem, researchers can use the cylindrical, as well as polar coordinate systems.

1. For different applications, various nanoparticles can be added.
2. Chemical processes and viscous dissipation can be added in the governing equation.
3. Extending the current study, it’s an excellent idea to include the diffusion-thermo and thermo-diffusion impacts in the governing equations.
4. Using other fractional derivatives, the described models can be solved.
5. To deal with the challenges, the Hankel transform technique can be used instead of the Laplace and sine Fourier transform approaches.
6. This problem can be extended to other non-Newtonian fluids, such as second-grade fluid, Jeffery fluid, Walter's-B fluid, Maxwell fluid and Oldroyd-B fluid.
7. It would also be useful if the reserachers in future studies focus on the size and morphology limitations in the model adapted.

## Data Availability

All data generated or analyzed during this study are included in this published article.

## List of symbol

*ρ*_*nf*_: Density of nanofluid
*β*^*^: Brinkman parameter
*σ*_*nf*_: Electrical conductivity of nanofluids
*g*: Acceleration due to gravity
$(\rho \beta_{c} )_{nf}$: Coefficient of concentration of nanofluid
*C*_1_: Dimentional concentration in $x$- direction
$$C_{\infty }$$: Constant concentration
$u_{1}$: Dimentional velocity in $x -$ direction
$\mu_{nf}$: Dynamic viscosity of nanofluid
$B_{0}$: Induced magnetic field
$(\rho \beta_{T} )_{nf}$: Thermal expansion coefficient of nanofluid
$$T_{\infty } :$$: Ambient temperature
$T_{1}$: Dimentional temperature in $x$-direction
*t*: Time

## References

[1] Neale G, Nader W (1974). Practical significance of brinkman’s extension of darcy’s law: Coupled parallel flows within a channel and a bounding porous medium. Can. J. Chem. Eng..

[2] Brinkman HC (1949). On the permeability of media consisting of closely packed porous particles. Appl. Sci. Res..

[3] Nazar R, Amin N, Filip D, Pop I (2003). The Brinkman model for the mixed convection boundary layer flow past a horizontal circular cylinder in a porous medium. Int. J. Heat Mass Transf..

[4] Changhao L, Payne LE (2007). Structural stability for a Brinkman fluid. Math. Methods Appl. Sci..

[5] Choi SUS, Eastman JA (1995). Enhancing thermal conductivity of fluids with nanoparticles. JAM.

[6] Murtaza S, Kumam P, Kaewkhao A, Khan N, Ahmad Z (2022). Fractal fractional analysis of non linear electro osmotic flow with cadmium telluride nanoparticles. Sci. Rep..

[7] Shah J (2022). MHD flow of time-fractional Casson nanofluid using generalized Fourier and Fick’s laws over an inclined channel with applications of gold nanoparticles. Sci. Rep..

[8] Khan N (2023). A time fractional model of a Maxwell nanofluid through a channel flow with applications in grease. Sci. Rep..

[9] Murtaza, S., Kumam, P., Ahmad, Z., Sittithakerngkiet, K. & Ali, I. E. Finite difference simulation of fractal-fractional model of electro-osmotic flow of casson fluid in a micro channel. in *IEEE Access* 1–1 (2022). doi:10.1109/ACCESS.2022.3148970.

[10] Saidur R, Leong KY, Mohammed HA (2011). A review on applications and challenges of nanofluids. Renew. Sustain. Energy Rev..

[11] Gorji TB, Ranjbar AA (2017). A review on optical properties and application of nanofluids in direct absorption solar collectors (DASCs). Renew. Sustain. Energy Rev..

[12] Sajid MU, Ali HM (2019). Recent advances in application of nanofluids in heat transfer devices: A critical review. Renew. Sustain. Energy Rev..

[13] Khan, H., Ali, F., Khan, N., Khan, I. & Mohamed, A. Electromagnetic flow of casson nanofluid over a vertical riga plate with ramped wall conditions. *Front. Phys.* 903 (2022).

[14] Timofeeva EV, Routbort JL, Singh D (2009). Particle shape effects on thermophysical properties of alumina nanofluids. J. Appl. Phys..

[15] Qi W, Liu QH (2005). Shape factor of nonspherical nanoparticles time-dependent perturbation theory in quantum mechanics view project. Artic. J. Mater. Sci..

[16] Ali F, Haq F, Khan N, Imtiaz A, Khan I (2022). A time fractional model of hemodynamic two-phase flow with heat conduction between blood and particles: Applications in health science. Waves Random Complex Media.

[17] Ali F (2022). A report of generalized blood flow model with heat conduction between blood and particles computational mathematics group project view project reliable numerical techniques for the solution of epidemic models with non-homogeneous population view project a report of generalized blood flow model with heat conduction between blood and particles. Artic. J. Magn..

[18] Meiorin C, Muraca D, Pirota KR, Aranguren MI, Mosiewicki MA (2014). Nanocomposites with superparamagnetic behavior based on a vegetable oil and magnetite nanoparticles. Eur. Polym. J..

[19] Link S, Burda C, Nikoobakht B, El-Sayed MA (2000). Laser-induced shape changes of colloidal gold nanorods using femtosecond and nanosecond laser pulses. J. Phys. Chem. B.

[20] Izgaliev AT, Simakin AV, Shafeev GA (2004). Formation of the alloy of Au and Ag nanoparticles upon laser irradiation of the mixture of their colloidal solutions. Kvantovaya Elektron..

[21] Hamilton RL (1962). Thermal conductivity of heterogeneous two-component systems. Ind. Eng. Chem. Fundam..

[22] Khan JA, Mustafa M, Hayat T, Turkyilmazoglu M, Alsaedi A (2017). Numerical study of nanofluid flow and heat transfer over a rotating disk using Buongiorno’s model. Int. J. Numer. Methods Heat Fluid Flow.

[23] Li D, Xie W, Fang W (2011). Preparation and properties of copper-oil-based nanofluids. Nanoscale Res. Lett..

[24] Fairuz, M., Adlina, M. N.,A. A.-A. M. Investigation of chip formation and tool wear in drilling process using various types of vegetable-oil based lubricants. *Trans Tech Publ*. (2015)

[25] Kuram E, Cetin MH, Ozcelik B, Demirbas E (2012). Performance analysis of developed vegetable-based cutting fluids by D-optimal experimental design in turning process. Int. J. Comput. Integr. Manuf..

[26] Khan, N. *et al.* Maxwell nanofluid flow over an infinite vertical plate with ramped and isothermal wall temperature and concentration. *Math. Probl. Eng.* **2021** (2021).

[27] Chandran P, Sacheti NC, Singh AK (2005). Natural convection near a vertical plate with ramped wall temperature. Heat Mass Transf..

[28] Khalid A, Khan I, Shafie S (2015). Exact solutions for free convection flow of nanofluids with ramped wall temperature. Eur. Phys. J. Plus.

[29] Ghara N, Das S, Maji SL, Jana RN (2012). Effect of radiation on MHD free convection flow past an impulsively moving vertical plate with ramped wall temperature. Am. J. Sci. Ind. Res..

[30] Hasin F, Ahmad Z, Ali F, Khan N, Khan I (2022). A time fractional model of Brinkman-type nanofluid with ramped wall temperature and concentration. Adv. Mech. Eng..

[31] Haq, S. U., Khan, I., Ali, F., Khan, A. & Abdelhameed, T. N. A. Influence of slip condition on unsteady free convection flow of viscous fluid with ramped wall temperature. *Abstr. Appl. Anal.* **2015**, (2015).

[32] Marneni N, Tippa S, Pendyala R (2015). Ramp temperature and Dufour effects on transient MHD natural convection flow past an infinite vertical plate in a porous medium. Eur. Phys. J. Plus.

[33] Anwar Beg O (2020). Computation of non-isothermal thermo-convective micropolar fluid dynamics in a hall MHD generator system with non-linear distending wall. Int. J. Appl. Comput. Math..

[34] Reddy PS, Sreedevi P, Chamkha AJ (2022). Hybrid nanofluid heat and mass transfer characteristics over a stretching/shrinking sheet with slip effects. J. Nanofluids.

[35] Reddy PS, Sreedevi P (2022). Effect of thermal radiation on heat transfer and entropy generation analysis of MHD hybrid nanofluid inside a square cavity. Waves Random Complex Media.

[36] Reddy PS, Sreedevi P, Chamkha AJ (2018). Magnetohydrodynamic (MHD) boundary layer heat and mass transfer characteristics of nanofluid over a vertical cone under convective boundary condition. Propuls. Power Res..

[37] Reddy PS, Sreedevi P, Reddy VN (2022). Entropy generation and heat transfer analysis of magnetic nanofluid flow inside a square cavity filled with carbon nanotubes. Chem. Thermodyn. Therm. Anal..

[38] Reddy PS, Sreedevi P, Chamkha AJ (2017). Thermodiffusion and diffusion &minus: Thermo effects on mhd heat and mass transfer of micropolar fluid over a stretching sheet. Int. J. Fluid Mech. Res..

[39] Sudarsana Reddy P, Sreedevi P (2023). Heat and mass transfer analysis of single walled carbon nanotubes-water and multi wall carbon nanotubes-water based maxwell nanofluid flow over stretchable rotating disks. J. Nanofluids.

[40] Khan N (2023). Dynamics of chaotic system based on circuit design with Ulam stability through fractal-fractional derivative with power law kernel. Sci. Rep..

[41] Ahmad Z, Ali F, Alqahtani AM, Khan N, Khan I (2021). Dynamics of cooperative reactions based on chemical kinetics with reaction speed: A comparative analysis with singular and nonsingular kernels. Fractals.

[42] Khan M (2023). Dynamics of two-step reversible enzymatic reaction under fractional derivative with Mittag–Leffler kernel. PLoS ONE.

[43] Ahmad Z, Ali F, Khan N, Khan I (2021). Dynamics of fractal-fractional model of a new chaotic system of integrated circuit with Mittag-Leffler kernel. Chaos Solitons Fractals.

[44] Khan N (2022). Dynamics of chaotic system based on image encryption through fractal-fractional operator of non-local kernel. AIP Adv..

[45] Kulish VV, Lage JL (2002). Application of fractional calculus to fluid mechanics. J. Fluids Eng..

[46] Podlubny I, Chechkin A, Skovranek T, Chen Y, Jara BMV (2009). Matrix approach to discrete fractional calculus II: Partial fractional differential equations. J. Comput. Phys..

[47] Ahmad Z (2022). Dynamics of love affair of romeo and juliet through modern mathematical tools: A critical analysis via fractal-fractional differential operator. Fractals.

[48] Ahmad Z, Arif M, Khan I (2020). Dynamics of fractional order SIR Model with a case study of COVID-19 in Turkey. City Univ. Int. J. Comput. Anal..

[49] Haq SU, Khan MA, Shah NA (2018). Analysis of magnetohydrodynamic flow of a fractional viscous fluid through a porous medium. Chin. J. Phys..

[50] Saqib M (2018). Exact solutions for free convection flow of generalized Jeffrey fluid: A Caputo–Fabrizio fractional model. Alex. Eng. J..

[51] Jamil M, Rauf A, Zafar AA, Khan NA (2011). New exact analytical solutions for Stokes’ first problem of Maxwell fluid with fractional derivative approach. Comput. Math. Appl..

[52] Tripathi D, Gupta PK, Das S (2011). Influence of slip condition on peristaltic transport of a viscoelastic fluid with fractional Burger’s model. Therm. Sci..

[53] Saqib M, Khan I, Shafie S, Mohamad AQ, Sherif ESM (2021). Analysis of magnetic resistive flow of generalized Brinkman type nanofluid containing carbon nanotubes with ramped heating. Comput. Mater Contin..

[54] Oztop HF, Abu-Nada E (2008). Numerical study of natural convection in partially heated rectangular enclosures filled with nanofluids. Int. J. Heat Fluid Flow.

[55] Ali F, Ahmad Z, Arif M, Khan I, Nisar KS (2020). A time fractional model of generalized couette flow of couple stress nanofluid with heat and mass transfer: Applications in engine oil. IEEE Access.

[56] Caputo M, Fabrizio M (2015). A new definition of fractional derivative without singular kernel. Prog. Fract. Differ. Appl..

